# Diversity and spatial distribution of malaria vectors in the WHO Eastern Mediterranean region from 1900 to 2024: a systematic review

**DOI:** 10.1186/s12936-025-05697-9

**Published:** 2025-11-29

**Authors:** Roya Rashti, Ebrahim Chavoshi, Leili Tapak, Fatemeh Nikpoor, Erfan Ayubi, Hossein Asakereh, Younes Mohammadi

**Affiliations:** 1 Department of Epidemiology, School of Public Health, Hamadan University of Medical Sciences, Hamadan, Iran; 2 Department of Environmental Health Engineering, School of Public Health, Hamadan University of Medical Sciences, Hamadan, Iran; 3 Department of Biostatistics, School of Public Health, Hamadan University of Medical Science, Hamadan, Iran; 4 Modeling of Noncommunicable Diseases Research Center, Institute of Health Sciences and Technologies, Hamadan University of Medical Sciences, Hamadan, Iran; 5 Department of Vector-Born Disease, Center for Communicable Disease Control, Ministry of Health, Tehran, Iran; 6 Social Determinants of Health Research Center, Institute of Health Sciences and Technologies, Avicenna Health Research Institute, Hamadan University of Medical Sciences, Hamadan, Iran; 7 Department of Geography, University of Zanjan, Zanjan, Iran

**Keywords:** *Anopheles* species, Diversity, Species distribution, Eastern Mediterranean region

## Abstract

**Background:**

Malaria remains a major public health challenge in the WHO Eastern Mediterranean region, transmitted by various *Anopheles* species. The main objective of this review was to collect the knowledge on the diversity and spatial distribution of malaria vectors in the World Health Organization (WHO) Eastern Mediterranean region.

**Methods:**

A comprehensive literature search was conducted in PubMed, Web of Science, and Scopus through August 2024 using the terms “*Anopheles*”, “malaria vector”, and relevant country names. Eligible field studies documenting captures of adult or larval *Anopheles* with detailed information about the capture locations (including both rural and urban areas, as well as various breeding sites and feeding behaviours), were systematically included. Out of 3637 identified publications, 276 met the inclusion criteria.

**Results:**

This systematic review identified 45 species of *Anopheles* mosquitoes documented across the WHO Eastern Mediterranean region, reflecting substantial ecological diversity. Most research has concentrated on five principal species: *Anopheles stephensi* (102 studies), *Anopheles superpictus* (57), *Anopheles dthali* (49), *Anopheles culicifacies* (47), and *Anopheles maculipennis* (45). A total of 18 countries were represented, with Iran contributing the largest share of studies (122 studies), followed by Pakistan (35), Sudan (31), Egypt (30), Morocco (14), Saudi Arabia (14), Iraq (7), Afghanistan (6), while the remaining countries had fewer than five studies each.

**Conclusions:**

This review highlights the critical need for ongoing monitoring and surveillance of *Anopheles* populations to identify shifts in distribution and abundance. Such information is essential for timely interventions and adapting control measures in response to emerging threats.

**Supplementary Information:**

The online version contains supplementary material available at 10.1186/s12936-025-05697-9.

## Background

Malaria is a serious and potentially life-threatening illness caused by parasitic organisms transmitted to humans through the bites of infected female *Anopheles* mosquitoes [1]. This disease is both preventable and treatable [2]. Five specific parasites are known to cause malaria in humans, with *Plasmodium falciparum* and *Plasmodium vivax* posing the greatest threat [3]. In 2023, it was estimated that there were approximately 263 million cases of malaria, resulting in an anticipated mortality of around 597,000 individuals [4]. Malaria incidence in the Eastern Mediterranean Region increased from 41.6 cases per 1000 in 2015 to 61.5 cases per 1000 in 2020, with notable increases in Djibouti, Somalia, Sudan, and Yemen following the detection of *Anopheles stephensi* [5]. Worldwide, 41 main species of mosquitoes can transmit malaria, with 9 species in the Americas, 6 in Europe and the Middle East, 7 in Africa, and 19 in Asia [6]. To prevent malaria, several control methods can be employed, including insecticide-treated nets (ITNs), indoor residual spraying (IRS), topical repellents, insecticide sprays, microbial larvicides, and home improvements, such as screening and insecticide wall coatings [7]. Larval source management, which often combines two or more of these methods, is also effective [8]. Political instability and population displacement can disrupt healthcare systems and malaria control programs, increasing vulnerability to outbreaks [9].

Many countries have been certified as having eliminated malaria between 1955 and 2024. Some of these countries implemented control measures, while others have been malaria-free from the start or witnessed the disease disappear without any intervention [10]. Previous studies have identified various species of *Anopheles* mosquitoes in the Eastern Mediterranean region [11–13]. However, comprehensive data on their distribution remains limited [5]. This study aims to gather information on the diversity and the geographical distribution of *Anopheles* across all Eastern Mediterranean region, without any time restrictions with a focus on the last decade. By simplifying this information and pinpointing the geographical locations of *Anopheles* populations, we can identify areas at potential risk for malaria [14]. This will aid in developing and implementing effective malaria prevention strategies, as well as in the strategic allocation of resources for disease control. Additionally, insights into vector distribution can assist researchers in creating new control methods and contribute to improving public health outcomes in affected countries [15, 16].

## Methods

### Study area

**Fig. 1:**
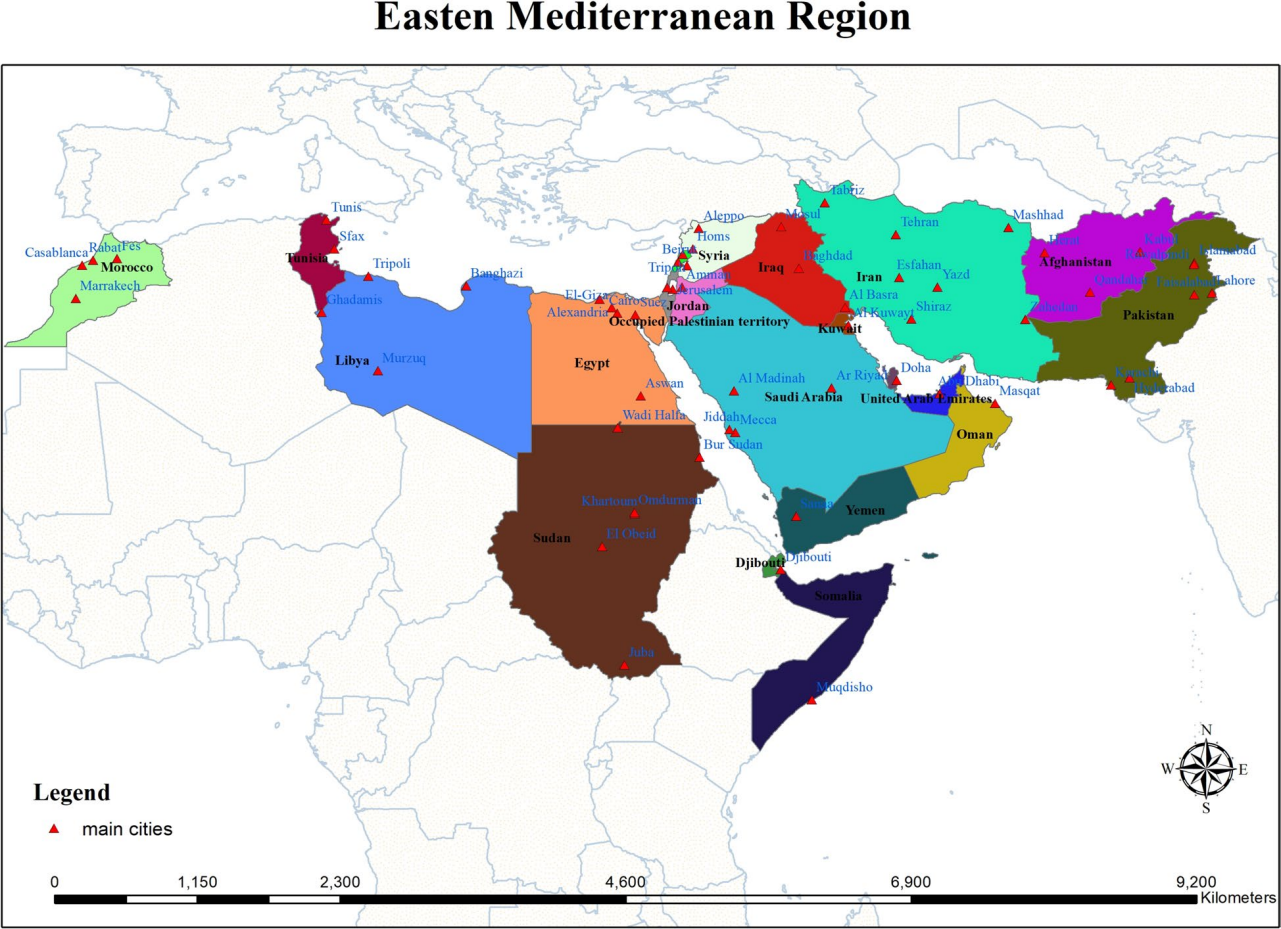
Geographic location of the WHO Eastern Mediterranean Region (study area)

This study was conducted in the World Health Organization (WHO) Eastern Mediterranean Region (EMR), which comprises 22 countries extending from North Africa and the Middle East to Central and South Asia [17]. The region covers diverse geographical landscapes, including arid deserts, coastal plains, mountain ranges, fertile river valleys, and tropical lowlands. The EMR experiences a wide range of climatic conditions, from hyper-arid desert climates in the Arabian Peninsula and North Africa to Mediterranean and subtropical climates in the Levant and mountainous regions, and monsoon-influenced environments in parts of Pakistan and Afghanistan [18]. The total population of the EMR is nearly 679 million people, characterized by a mixture of densely populated urban centers, rapidly growing peri-urban areas, and rural communities [19]. Environmental and ecological conditions vary substantially across the region, influencing mosquito breeding habitats and malaria transmission dynamics [20]. Key ecological features include oases and irrigation systems, river basins such as the Nile and Indus, coastal wetlands, and temporary rain-dependent water bodies [21]. Figure 1 shows the geographic extent of the WHO Eastern Mediterranean Region, including the countries included in this review.

### PICOTS criteria

- Population: countries of the WHO Eastern Mediterranean region
- Intervention (exposure): not applicable
- Control: not applicable
- Outcome: Presence of malaria vectors
- Time: 1900 to 2024
- Study: field study

### Eligibility criteria

The documents selected for this study were based on the eligibility criteria. The inclusion criteria were: field studies that recorded the capture of either the adult insect or its larvae, along with exact information about the capture location (including any rural or urban areas, any breeding sites, and any feeding habits), were selected. Studies were excluded if they: (1) were not conducted in countries of the WHO Eastern Mediterranean Region; (2) did not report field collection of *Anopheles* mosquitoes (either adults or larvae); (3) lacked specific geographic location data; (4) were laboratory-based studies without field sampling; (5) lacked sufficient methodological information.

### Search strategy

The search for articles was conducted in the online databases like PubMed, Web of Science, and Scopus. These databases were systematically examined up to August 20, 2024. Additionally, the reference lists of the studies included in this review were explored. The following combinations of search terms were used: "*Anopheles*" OR "Malaria vector" AND "Afghanistan" OR "Bahrain" OR "Djibouti" OR "Egypt" OR "Iran" OR "Iraq" OR "Jordan" OR "Kuwait" OR "Lebanon" OR "Libya" OR "Morocco" OR "Palestine" OR "Oman" OR "Pakistan" OR "Qatar" OR "Saudi Arabia" OR "Somalia" OR "Sudan" OR "Syrian Arab Republic" OR "Tunisia" OR "United Arab Emirates" OR "Yemen" (Additional file 1: Table S1).

### Study selection

The search results from all databases were compiled using EndNote X8, and duplicate entries were systematically eliminated. Following this, two authors (R.R. and F.N.) independently reviewed the titles and abstracts to exclude studies that did not meet the eligibility criteria. Any disagreements were resolved with the assistance of a third person (Y.M.). The full texts of studies considered potentially relevant were then retrieved for a thorough evaluation.

### Data extraction

Two researchers (R.R. and Y.M.) independently extracted the study data using a structured electronic form, designed and implemented in the Excel software version 2021. The gathered data included: the name of the author, the year in which the work was published, the year of the study was conducted, the species of *Anopheles*, the name of the country, province, city, and village, as well as the geographic coordinates of the locations where the *Anopheles* mosquito is documented to exist (in instances where the geographic coordinates are not provided within the manuscript, this information was obtained from Google Maps). Moreover, all maps were made using ArcGIS 10.8.2.

## Results

### Search results

**Fig. 2:**
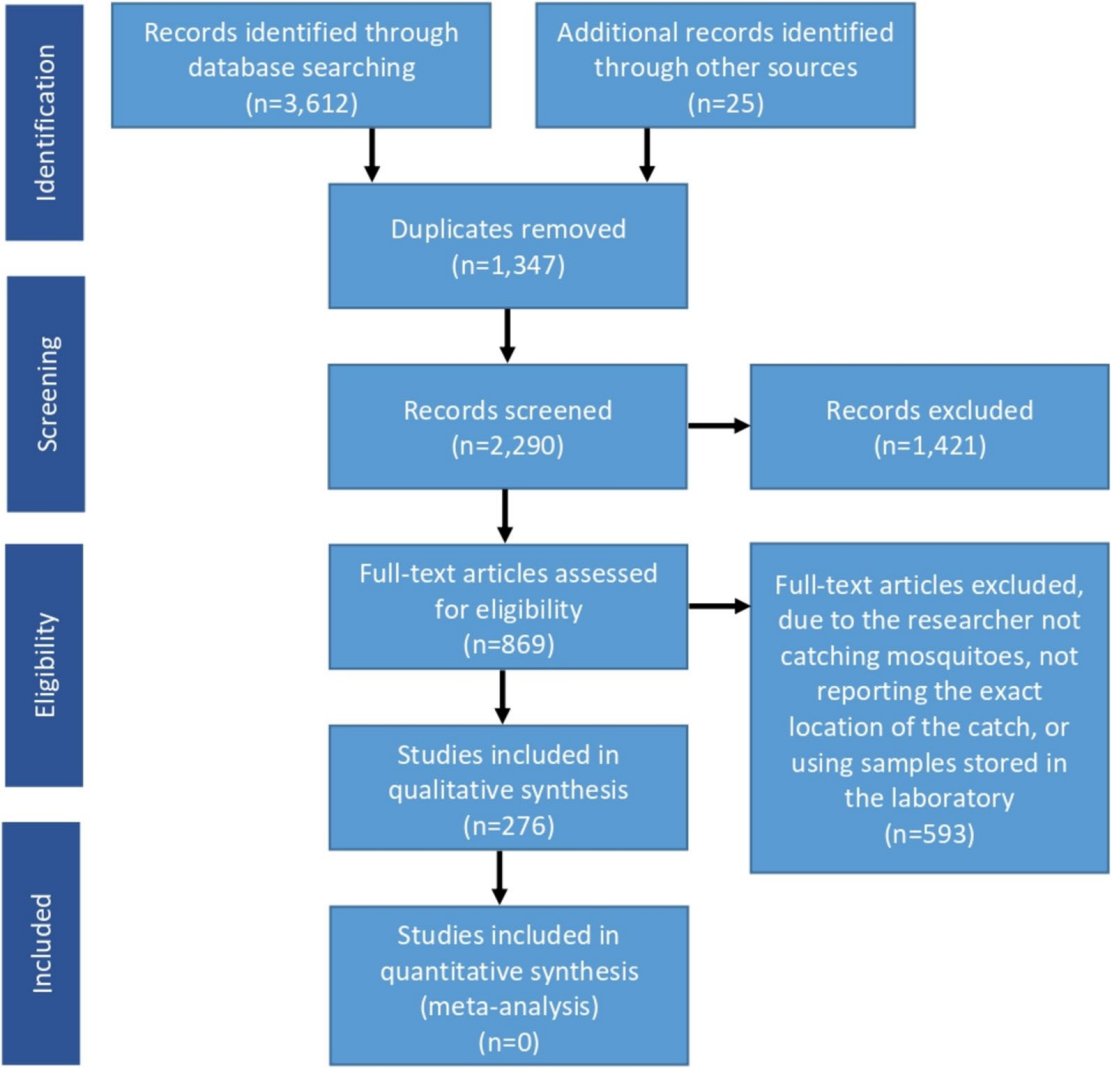
Flow of information through the various phases of the systematic review

After analysing the different databases, 3637 scholarly articles were compiled. After eliminating duplicates and conducting a careful review of titles and abstracts, 869 records were selected for an in-depth evaluation of their full texts. Ultimately, 276 articles were deemed to fully meet the established inclusion criteria (Fig. 2).

### Study characteristics

**Fig. 3:**
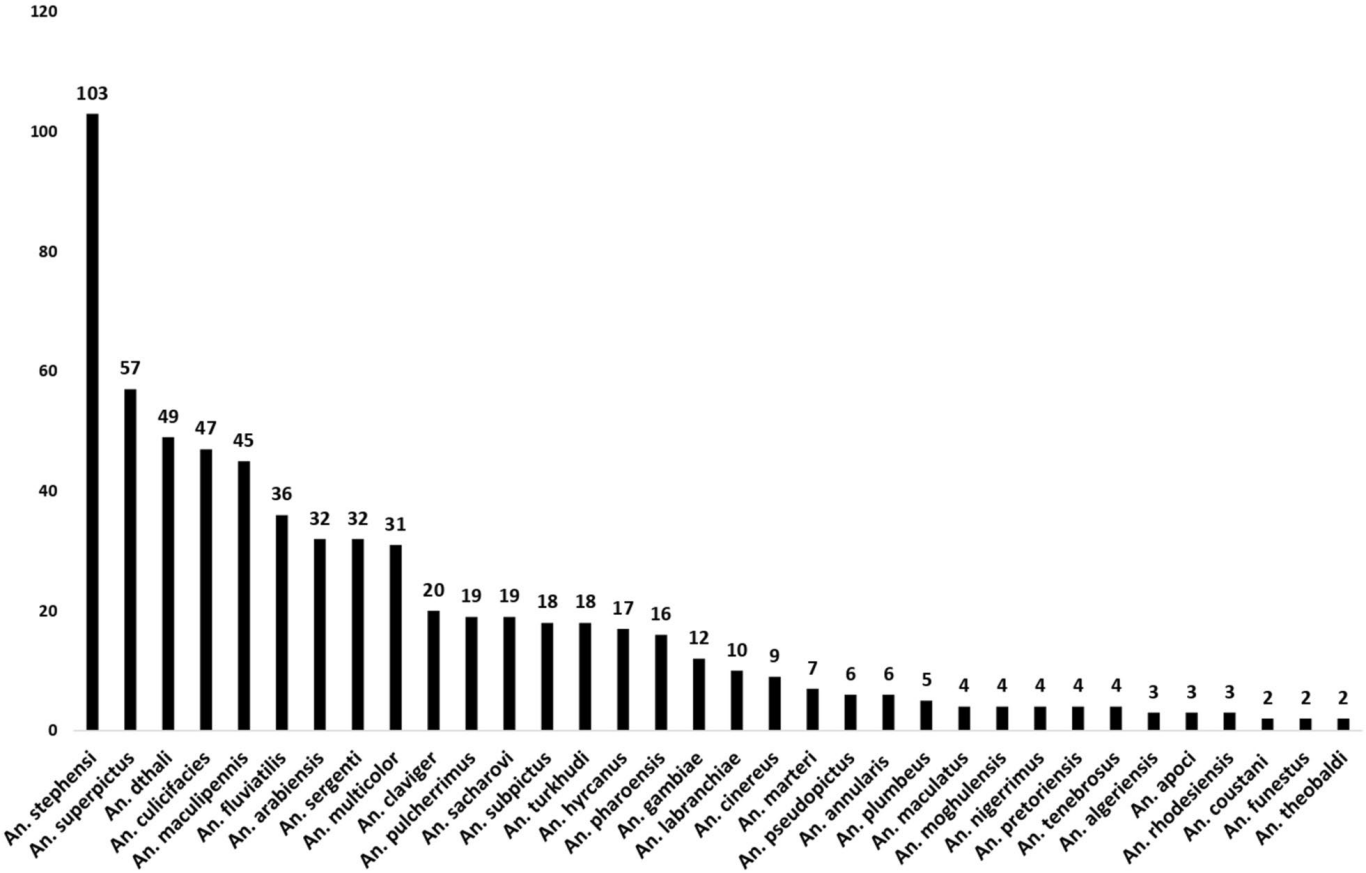
Chart of the number of studies that report the presence of a species

The characteristics of the studies included in this research can be found in Additional file 2: Table S2. A total of 276 studies documented the presence of 45 *Anopheles* species across countries in the WHO Eastern Mediterranean region. The majority of these studies focused on five species: *Anopheles stephensi* (102 studies), *Anopheles superpictus* (57), *Anopheles dthali* (49), *Anopheles culicifacies* (47), and *Anopheles maculipennis* (45) (Fig. 3). Additionally, the species *Anopheles azaniae*, *Anopheles hispaniola*, *Anopheles lindesayi*, *Anopheles minimus*, *Anopheles myzomyia*, *Anopheles pallidus*, *Anopheles polacrimus*, *Anopheles rufipes, Anopheles rupicolus, Anopheles splendidus,* and *Anopheles tesselatus* have each been reported in only one study.

The studies were from 18 countries. The highest number of studies originated from Iran (122), followed by Pakistan (35), Sudan (31), Egypt (30), Morocco (14), Saudi Arabia (14), Iraq (7), and Afghanistan (6). The remaining countries each contributed fewer than five studies.

### Spatial distribution of *Anopheles* species in the WHO Eastern Mediterranean

**Fig. 4:**
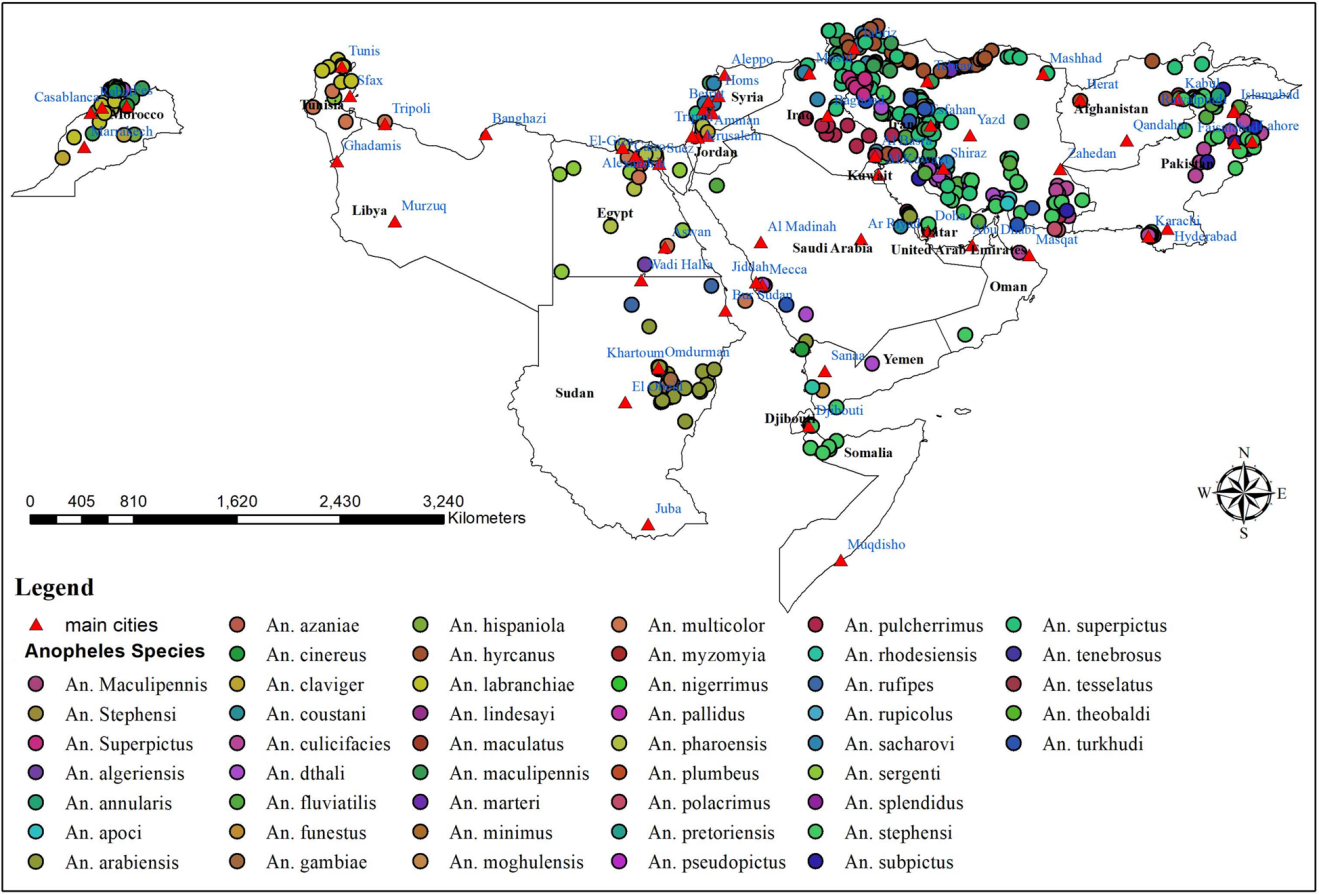
Spatial distribution of *Anopheles* species in the WHO Eastern Mediterranean Region, from 1900 to 2024

The distribution of *Anopheles* species in the WHO Eastern Mediterranean region from 1900 to 2024 is shown in Fig. 4. The specific distribution patterns of each *Anopheles* species are detailed as follows:

*Anopheles stephensi* has been documented in several regions, including the eastern parts of Afghanistan, the southern areas of Djibouti, central and southeastern Egypt, and throughout the southern, southeastern, and southwestern regions of Iran.

Additionally, it has been reported in the central and southeastern areas of Iraq, the northern and southern regions of Oman, as well as the northwest, northeast, east, and southeast of Pakistan. The species is also found in the northeastern part of Qatar, along with both western and eastern regions of Saudi Arabia, the northern areas of Somalia, and the southern parts of Yemen.

*Anopheles superpictus* has been documented in the eastern regions of Afghanistan, throughout Iran, the eastern and northeastern parts of Iraq, the northern and northwestern areas of Jordan, western Lebanon, the eastern and southeastern regions of Pakistan, southern Saudi Arabia, and western Syria.

*Anopheles dthali* has been observed in the southern and southeastern regions of Iran, as well as throughout the west, east, southwest, and southeast of Saudi Arabia.

Additionally, its presence has been documented in southern Jordan, northwest Morocco, southern Oman, northwest Pakistan, and east-central Yemen, with individual research studies conducted for each of these locations.

*Anopheles culicifacies*. The distribution of this species has been documented in several geographical regions, including the northwest, northeast, and southeastern parts of Pakistan. Additionally, its presence has been noted in the southern and southeastern regions of Iran, the northern territory of Oman, the eastern areas of Afghanistan, and the western and southwestern zones of Saudi Arabia, as well as in the western regions of Yemen.

*Anopheles maculipennis* has been recorded in northern, northwestern, and western Iran, as well as in northern, central, and northwestern Morocco.

Additionally, individual research studies have documented its presence in northern Iraq, southern Lebanon, and western Syria.

*Anopheles fluviatilis* has been recorded in various geographical regions, specifically within the southern and southeastern territories of Iran, as well as the eastern and western areas of Saudi Arabia.

Additionally, its presence has been noted in the northwestern and northeastern regions of Pakistan. Furthermore, a study has documented occurrences of this species in the eastern region of Afghanistan.

*Anopheles arabiensis* has been recorded in southern and southwestern Saudi Arabia, nearly all regions of Sudan, and Yemen, particularly in Zubaid city.

*Anopheles sergenti*. Reports of *An. sergenti* have been documented in various regions, including the north, northeast, central, and southeast of Egypt; the south of Iran; the northwest of Jordan; the northwest of Morocco; the south of Oman; the east and southwest of Saudi Arabia; the central part of Tunisia; and the east of Yemen.

*Anopheles multicolor* has been reported across various locations, including all regions of Egypt, the central and southern parts of Iran, northwest Jordan, and the Jordan Valley.

It can also be found in the eastern, western, and southwestern regions of Saudi Arabia, as well as in the central and southern areas of Tunisia. Additionally, this species has been documented in western Libya and at the Al Hassan Addakhil dam in Morocco, with individual research studies conducted for each of these locations.

*Anopheles claviger* has been observed in the northern, northwestern, and western regions of Iran.

Additionally, it has been documented in the eastern part of Lebanon (Beirut), southern Lebanon, the northern areas of Jordan (specifically in Jarash and Irbid Governorate), and the northeastern region of Morocco (Chefchaouen) through individual research studies conducted for each location.

*Anopheles pulcherrimus* has been observed in northeastern, eastern, southeastern, and southern Iran; northern Afghanistan; central, southern, and eastern Iraq; northeastern and northwestern Pakistan.

Additionally, it has also been identified in a study conducted in the eastern province of Saudi Arabia.

*Anopheles sacharovi* has been recorded in the southern, northern, and northwestern regions of Iran, as well as in the northern, eastern, and southeastern areas of Iraq, and in northern Jordan.

Additionally, it has been documented in northern Lebanon and western Syria through individual research studies for each of these locations.

*Anopheles subpictus* has been documented in the northwest, northeast, and southern areas of Pakistan, southern Iran, and across various regions of Saudi Arabia, including the west, east, southwest, and southeast.

*Anopheles turkhudi* has been documented in southern, central, and southeastern Iran, as well as southern Saudi Arabia.

*Anopheles hyrcanus* has been documented in northern, northeastern, and northwestern Iran, as well as northern and western Afghanistan, with a notable record also in the Jordan Valley.

*Anopheles pharoensis* has been documented in northeastern Egypt (both Lower and Upper regions) and central Egypt, as well as in eastern Sudan and northwest Jordan through individual research studies.

*Anopheles gambiae* has been documented in the eastern, east-central, and northeastern regions of Sudan, as well as the eastern, western, and southwestern areas of Saudi Arabia.

It has also been recorded in southern Djibouti and along Egyptian railways from upper to lower regions.

*Anopheles labranchiae* has been documented in the northern and northwestern regions of Morocco and Tunisia.

*Anopheles cinereus* has been reported in eastern, southern, and western Saudi Arabia, and in northern and northeastern Morocco.

*Anopheles marteri* has been recorded in the northern, central, and western parts of Iran.

It has also been documented in a singular study conducted in the northwest region of Jordan, specifically within Ajloun Province.

## *Anopheles apoci, Anopheles pseudopictus, Anopheles moghulensis, Anopheles plumbeus,* and *Anopheles polacrimus*

These *Anopheles* species have been reported exclusively in Iran. *Anopheles apoci*, *An. moghulensis*, and *An. polacrimus* have been identified in the southern regions. *Anopheles* pseudopictus was observed in the northern and northwestern areas. Additionally, *An. plumbeus* has been noted in the northern region of Iran.

*Anopheles annularis, Anopheles nigerrimus, Anopheles pallidus, Anopheles lindesayi, Anopheles maculatus, Anopheles splendidus, Anopheles tesselatus, and Anopheles theobaldi*. These *Anopheles* species have been documented exclusively in the northwestern and northeastern regions of Pakistan, specifically within the provinces of Punjab and Khyber Pakhtunkhwa.

*Anopheles azaniae, Anopheles pretoriensis and Anopheles rupicolus*. These Anopheles species have been exclusively reported in Saudi Arabia. Anopheles azaniae is primarily found in the Jezan region, while An. pretoriensis has been documented in the eastern, southern, and southwestern areas of the country. Moreover, An. rupicolus has been observed in the southern region of Saudi Arabia.

*Anopheles myzomyia* and *Anopheles rufipes. *The following *Anopheles* species have been definitively reported only in Sudan. *Anopheles myzomyia* is found in the southeastern region, while *An. rufipes* is prominently located in the north.

*Anopheles algeriensis* has been documented in northwestern Jordan, southern Egypt, and southwestern Tunisia.

*Anopheles coustani* has been documented in a study conducted in the eastern regions of Saudi Arabia, as well as in another investigation focusing on the northern regions of Egypt.

*Anopheles hispaniola* was documented in a singular study conducted at the Al Hassan Addakhil Dam in Morocco.

*Anopheles tenebrosus* has been documented in the eastern region of Saudi Arabia and the northern territory of Egypt.

*Anopheles funestus* has been documented from the east of Sudan and the south of the Yemen in an individual research study for each location.

*Anopheles rhodesiensis* has been reported from the Tehama area, west of Yemen and east of Saudi Arabia.

**Fig. 5:**
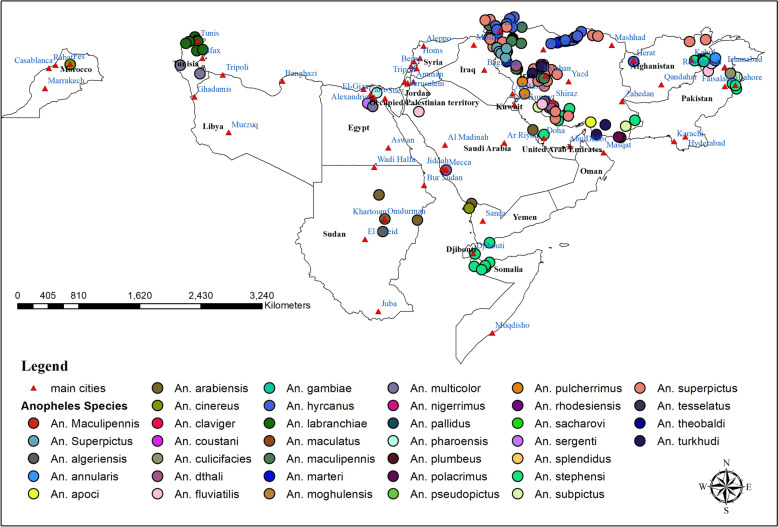
Spatial distribution of *Anopheles* species in the WHO Eastern Mediterranean Region, from 2015 to 2024

A comparison of *Anopheles* species reported in WHO Eastern Mediterranean countries from 1900 to 2024 and 2015 to 2024 (the last 10 years) is shown in Table 1. The distribution of *Anopheles* species reported from 2015 to 2024 is also shown in Fig. 5. The majority of reports continue to be from Iran, Pakistan, and Saudi Arabia. Iran has maintained the presence of all twenty (20) *Anopheles* species from 1900 to 2024. No study was found for Iraq, Jordan, Lebanon, Oman, Djibouti, and Syria. The presence of *An. stephensi* has also been seen in most countries in the last decade.

**Table 1:** The comparison of *Anopheles* species reported in Eastern Mediterranean Countries from 1900 to 2024 and 2015 to 2024 (the last ten years)

Country	1900–2024				2015–2024			
	No. of studies	No. of locations	No. of *Anopheles* species	*Anopheles* species	No. of studies	No. of locations	No. of *Anopheles* species	species
Iran	122	659	20	*An. apoci, An. claviger, An. culicifacies, An. dthali, An. fluviatilis, An. hyrcanus, An. maculipennis, An. marteri, An. moghulensis, An. multicolor, An. plumbeus, An. polacrimus, An. pseudopictus, An. pulcherrimus, An. sacharovi, An. sergenti, An. stephensi, An. subpictus, An. superpictus, An. turkhudi*	39	335	20	*An. apoci, An. claviger, An. culicifacies, An. dthali, An. fluviatilis, An. hyrcanus, An. maculipennis, An. marteri, An. moghulensis, An. multicolor, An. plumbeus, An. polacrimus, An. pseudopictus, An. pulcherrimus, An. sacharovi, An. sergenti, An. stephensi, An. subpictus, An. superpictus, An. turkhudi*
Pakistan	35	167	15	*An. annularis, An. culicifacies, An. dthali, An. fluviatilis, An. lindesayi, An. maculatus, An. nigerrimus, An. pallidus, An. pulcherrimus, An. splendidus, An. stephensi, An. subpictus, An. superpictus, An. tesselatus, An. theobaldi*	6	40	12	*An. annularis, An. culicifacies, An. dthali, An. fluviatilis, An. maculatus, An. nigerrimus, An. pallidus, An. splendidus, An. stephensi, An. subpictus, An. tesselatus, An. theobaldi*
Saudi Arabia	31	159	19	*An. arabiensis, An. azaniae, An. cinereus, An. coustani, An. culicifacies, An. dthali, An. fluviatilis, An. gambiae, An. multicolor, An. pretoriensis, An. pulcherrimus, An. rhodesiensis, An. rupicolus, An. sergenti, An. stephensi, An. subpictus, An. superpictus, An. tenebrosus, An. turkhudi*	6	19	8	*An. arabiensis, An. cinereus, An. dthali, An. fluviatilis, An. multicolor, An. rhodesiensis, An. sergenti, An. stephensi*
Sudan	30	93	6	*An. arabiensis, An. funestus, An. gambiae, An. myzomyia, An. pharoensis, An. rufipes*	4	10	2	*An. arabiensis, An. gambiae*
Moroco	14	74	8	*An. cinereus, An. claviger, An. dthali, An. hispaniola, An. labranchiae, An. maculipennis, An. multicolor, An. sergenti*	3	9	4	*An. cinereus, An. labranchiae, An. maculipennis, An. sergenti*
Egypt	14	69	8	*An. algeriensis, An. coustani, An. gambiae, An. multicolor, An. pharoensis, An. sergenti, An. stephensi, An. tenebrosus*	4	12	4	*An. coustani, An. multicolor, An. pharoensis, An. sergenti*
Iraq	7	37	5	*An. maculipennis An. pulcherrimus An. sacharovi An. stephensi An. superpictus*	0	0	0	NR
Afghanistan	6	19	6	*An. culicifacies, An. fluviatilis, An. hyrcanus, An. pulcherrimus, An. stephensi, An. superpictus*	2	12	3	*An. hyrcanus, An. stephensi, An. superpictus*
Jordan	4	18	10	*An. algeriensis, An. claviger, An. dthali, An. hyrcanus, An. marteri, An. multicolor, An. pharoensis, An. sacharovi, An. sergenti, An. superpictus*	0	0	0	NR
Tunisia	3	15	4	*An. algeriensis, An. labranchiae, An. multicolor, An. sergenti*	1	11	4	*An. algeriensis, An. labranchiae, An. multicolor, An. sergenti*
Lebanon	3	12	4	*An. claviger, An. maculipennis, An. sacharovi, An. superpictus*	0	0	0	NR
Yemen	2	8	8	*An. arabiensis, An. culicifacies, An. dthali, An. funestus, An. minimus, An. rhodesiensis, An. sergenti, An. stephensi*	1	1	1	*An. stephensi*
Somalia	2	6	1	*An. stephensi*	1	6	1	*An. stephensi*
Oman	2	5	4	*An. culicifacies An. dthali An. sergenti An. stephensi*	0	0	0	NR
Djibouti	2	3	2	*An. gambiae, An. stephensi*	0	0	0	NR
Syria	2	3	3	*An. maculipennis, An. sacharovi, An. superpictus*	0	0	0	NR
Qatar	1	2	1	*An. stephensi*	1	2	1	*An. stephensi*
Libya	1	1	1	*An. multicolor*	0	0	0	NR

*NR* not reported

## Discussion

This systematic review examines the diversity and spatial distribution of *Anopheles* species in the WHO Eastern Mediterranean Region from 1900 to 2024, as well as the last 10 years, revealing a complex assemblage of mosquito vectors with distinct geographic distributions that underscore the ecological diversity of the region. The spatial distribution of *Anopheles* species across the WHO Eastern Mediterranean is heterogeneous, with significant concentrations of these species in Iran, Pakistan, and Sudan. The high number of studies from Iran (n = 122) reflects the significant malaria burden in the country and ongoing efforts to monitor and control vector populations [9, 22]. In this study, *An. stephensi*, a known urban malaria vector, has been reported in a wide range of locations from Afghanistan to Yemen, indicating its adaptability to different environments [23]. The presence of *An. stephensi* in most studies over the past 10 years also suggests a role for this species in malaria transmission, highlighting the importance of ongoing surveillance and vector control efforts in the region. It is the most common *Anopheles* species in the WHO Eastern Mediterranean region, which also had the highest documented record in this study [24]. This species has spread from Asian countries and the Arabian Peninsula to the Horn of Africa. In other studies, the spreadability of *An. stephensi* has been mentioned in the absence of control [25, 26]. This adaptation poses a significant challenge to malaria control, especially in urban areas where the risk of transmission may be increased due to human-mosquito interactions [26]. In this study, species such as *An. culicifacies* and *An. superpictus* are more frequently encountered in Pakistan, Iran, and Saudi Arabia. These species are typically found in rural and agricultural environments [27–29]. These findings indicate that the dynamics of malaria transmission could vary greatly between urban and rural regions, necessitating tailored vector control measures that consider the specific ecological conditions of each area. The distribution patterns also reveal that certain species are restricted to specific geographic areas, such as *An. azaniae* and *An. pretoriensis* in Saudi Arabia, and *An. myzomyia* and *An. rufipes* in Sudan. This localized presence may be influenced by environmental factors such as climate, land use, and human activities, which can affect mosquito breeding habitats and survival rates. Understanding these factors is crucial for developing targeted vector control interventions [30, 31]. The identification of *An. gambiae* and *An. funestus* in regions like Sudan and Yemen, indeed raises concerns about increased malaria transmission due to their high efficiency as vectors. Both species are highly anthropophilic, meaning they prefer feeding on humans, which significantly enhances their role in malaria transmission [9]. Also, *An. funestus* has shown resistance to common insecticides like pyrethroids, which are widely used in insecticide-treated nets (ITNs) and indoor residual spraying (IRS) [32]. The presence of these species, belonging to two genera, in the region may complicate existing malaria control efforts and necessitate a reevaluation of current strategies.

In this study, several species of *Anopheles* have been reported in one study. The limited distribution of these species suggests that they may be under-researched [33], as in this study, some countries, such as Somalia and Libya, do not have sufficient documentation for the presence of *Anopheles* mosquitoes, and other studies have also pointed out this difference in the focus of research and the availability of data in different countries [13, 33], or that control measures have been implemented to combat them [34]. However, considering that some of these species, such as *An. pallidus, An. polacrimus, An. rufipes,* and *An. tesselatus,* in regions where they were not previously recorded (were published after 2000), may indicate changing ecological dynamics or the introduction of new vectors. For example, *An. pallidus* was recorded for the first time in the Garhwal region of India, suggesting localized climate-driven distribution changes [35]. Similarly, *An. tesselatus* was identified as a new malaria vector in South Kalimantan, Indonesia, highlighting its potential role in disease transmission in previously unaffected areas [36]. These findings underscore the importance of monitoring vector species, as their emergence or introduction could be linked to environmental changes, human activities, or shifts in vector control strategies. Further studies are needed to assess their ecological roles and potential impacts on public health.

There was no report of any *Anopheles* species in Iraq, Jordan, Lebanon, Oman, Djibouti, and Syria during the last 10 years (2015–2024), indicating a potential absence or lack of data on these species in that period. This may warrant further investigation into the ecological and environmental factors affecting *Anopheles* populations in the region [37]. The continuity of some *Anopheles* species in Iran, Pakistan, Saudi Arabia, and other countries from 1900 to 2024 highlights the importance of ongoing surveillance and research to understand their ecology and the potential impact on public health, particularly concerning vector-borne diseases.

One of the limitations of this study was the lack of investigation of the prevalence of *Anopheles* species. This is because we were looking for the locations of species presence and many studies had not investigated prevalence. They were genetic studies, insecticide studies, but they were included in this study because they had carried out mosquito trapping and reported the location of the trap. Also, this study did not investigate their feeding behaviours (anthropophilic, endophagous, endophilic or exophilic), habitat preferences (such as swamps, rivers, water reservoirs, and tanks), and transmission dynamics. These factors are crucial for the effective implementation of control measures.

## Conclusion

This systematic review aimed to identify and map the diversity and spatial distribution of *Anopheles* species across the WHO Eastern Mediterranean Region from 1900 to 2024. The findings demonstrated the presence of 45 *Anopheles* species in the region, with the highest number of reports from Iran, Pakistan, Saudi Arabia, and Sudan. *Anopheles stephensi* was the most frequently reported species, highlighting its expanding geographic range and increasing public health relevance. The heterogeneous distribution of vector species across diverse ecological settings underscores the importance of continuous entomological surveillance and adaptive malaria control strategies. Future research should focus on assessing the abundance and seasonal dynamics of *Anopheles* species, as well as their trophic behaviour and habitat preferences, to improve early detection and inform targeted vector management programmes. Strengthening integrated vector surveillance systems will be essential for mitigating the risk of malaria transmission in the Eastern Mediterranean Region.

## Supplementary Information

Additional file 1: Table S1: Search Strategy.

Additional file 2: Table S2: Characteristics of the Studies Included in this Research.

## Data Availability

The data used in this study must be available within the article and/or its supplementary materials, or deposited in a publicly available database.

## Abbreviations

IRS: Indoor residual spraying
ITNs: Insecticide-treated nets
PICOTS: Population, intervention, control, outcome, time, study
WHO: World Health Organization
