# Supplementary material for: Diversity and spatial distribution of malaria vectors in the WHO Eastern Mediterranean region from 1900 to 2024: a systematic review
Source: Malar J. 2025 Nov 29;25:8. doi: 10.1186/s12936-025-05697-9 (PMC12771993; doi:10.1186/s12936-025-05697-9)
Supplement: Supplementary file 1 — Additional file 1: Table S1: Search Strategy. [file 12936_2025_5697_MOESM1_ESM.docx]

Appendix 1: Search strategies.

| Databases, time of searches, and search terms | | Hits |  |
| --- | --- | --- | --- |
| Pubmed up to 20 August 2024 | | | |
| **#1** | ***Anopheles*[Text Word]** | [19,744](https://pubmed.ncbi.nlm.nih.gov/?term=Anopheles%5BText+Word%5D&sort=date) |  |
| **#2** | **"Malaria vector"[Text Word]** | [3,527](https://pubmed.ncbi.nlm.nih.gov/?term=%22malaria+vector%22%5BText+Word%5D&sort=date) |  |
| **#3** | **#1 OR #2** | 20,052 |  |
| **#4** | **Afghanistan** | 9,859 |  |
| **#5** | **Bahrain** | 5,190 |  |
| **#6** | **Djibouti** | 655 |  |
| **#7** | **Egypt** | 155,019 |  |
| **#8** | **Iran** | 288,752 |  |
| **#9** | **Iraq** | 27,022 |  |
| **#10** | **Jordan** | 55,739 |  |
| **#11** | **Kuwait** | 16,081 |  |
| **#12** | **Lebanon** | 45,457 |  |
| **#13** | **Libya** | 4,058 |  |
| **#14** | **Morocco** | 25,979 |  |
| **#15** | **Palestine** | 5,975 |  |
| **#16** | **Oman** | 15,764 |  |
| **#17** | **Pakistan** | 108,385 |  |
| **#18** | **Qatar** | 23,755 |  |
| **#19** | **"Saudi Arabia"** | 166,753 |  |
| **#20** | **Somalia** | 3,452 |  |
| **#21** | **Sudan** | 17,644 |  |
| **#22** | **"Syrian Arab Republic"** | 673 |  |
| **#23** | **Tunisia** | 34,592 |  |
| **#24** | **"United Arab Emirates"** | 28,622 |  |
| **#25** | **Yemen** | 5,657 |  |
| **#26** | **#4 OR #5 OR #6 OR #7 OR #8 OR #9 OR #10 OR #11OR #12OR #13 OR #14 OR #15 OR #16 OR #17 OR #18 OR #19 OR #20 OR #21 OR #22 OR #23 OR #24 OR #25 OR #26** | 927,742 |  |
| **#27** | **#3 AND #26** | 1,008 |  |
| Web of science up to 20 August 2024 | | | |
| **#1** | **(TS=(*Anopheles*)) OR TS=("Malaria vector")** | 21,304 |  |
| **#2** | **(((((((((((((((((((((TS=(Afghanistan)) OR TS=(Bahrain)) OR TS=(Djibouti)) OR TS=(Egypt)) OR TS=(Iran)) OR TS=(Iraq)) OR TS=(Jordan)) OR TS=(Kuwait)) OR TS=(Lebanon)) OR TS=(Libya)) OR TS=(Morocco)) OR TS=(Palestine)) OR TS=(Oman)) OR TS=(Pakistan)) OR TS=(Qatar)) OR TS=("Saudi Arabia")) OR TS=(Somalia)) OR TS=(Sudan)) OR TS=("Syrian Arab Republic")) OR TS=(Tunisia)) OR TS=("United Arab Emirates")) OR TS=(Yemen)** | 525,351 |  |
| **#3** | **#1 AND #2** | 676 |  |
| Scopus up to 20 August 2024 | |  |  |
| **#1** | **( TITLE-ABS-KEY ( *Anopheles* ) ) OR ( TITLE-ABS-KEY ( "Malaria vector" ) )** | 25,772 |  |
| **#2** | **( TITLE-ABS-KEY ( Afghanistan ) ) OR ( TITLE-ABS-KEY ( Bahrain ) ) OR ( TITLE-ABS-KEY ( Djibouti ) ) OR ( TITLE-ABS-KEY ( Egypt ) ) OR ( TITLE-ABS-KEY ( Iran ) ) OR ( TITLE-ABS-KEY ( Iraq ) ) OR ( TITLE-ABS-KEY ( Jordan ) ) OR ( TITLE-ABS-KEY ( Kuwait ) ) OR ( TITLE-ABS-KEY ( Lebanon ) ) OR ( TITLE-ABS-KEY ( Libya ) ) OR ( TITLE-ABS-KEY ( Morocco ) ) OR ( TITLE-ABS-KEY ( Palestine ) ) OR ( TITLE-ABS-KEY ( Oman ) ) OR ( TITLE-ABS-KEY ( Pakistan ) ) OR ( TITLE-ABS-KEY ( Qatar ) ) OR ( TITLE-ABS-KEY ( "Saudi Arabia" ) ) OR ( TITLE-ABS-KEY ( Somalia ) ) OR ( TITLE-ABS-KEY ( Sudan ) ) OR ( TITLE-ABS-KEY ( "Syrian Arab Republic" ) ) OR ( TITLE-ABS-KEY ( Tunisia ) ) OR ( TITLE-ABS-KEY ( "United Arab Emirates" ) ) OR ( TITLE-ABS-KEY ( Yemen ) )** | 796,116 |  |
| **#3** | **#1 AND #2** | 965 |  |
| Total | | 3612 |  |
| Duplicates | | 1347 |  |
| Final | | 2,265 |  |
