# Supplementary material for: Diversity and spatial distribution of malaria vectors in the WHO Eastern Mediterranean region from 1900 to 2024: a systematic review
Source: Malar J. 2025 Nov 29;25:8. doi: 10.1186/s12936-025-05697-9 (PMC12771993; doi:10.1186/s12936-025-05697-9)
Supplement: Supplementary file 2 — Additional file 2: Table S2: Characteristics of the Studies Included in this Research. [file 12936_2025_5697_MOESM2_ESM.docx]

Supplementary file 1: the Characteristics of the Studies Included in this Research.

| **Author**  **(Ref)** | **Year of publication** | **Year of assessment** | **Country** | **Province/city/village** | **Type of *Anopheles*** |  |
| --- | --- | --- | --- | --- | --- | --- |
| Büttiker, W.  (1) | 1958 | 1956 | Afghanistan | Jalalabad | *An. culicifacies* |  |
| Dukhanina, N. N.  (2) | 1975 |  | Afghanistan | Aryk Khazara-Tugay | *An. pulcherrimus, An. hyrcanus* |  |
| Onori, E.  (3) | 1975 | 1973 | Afghanistan | Kunduz; Quchi | *An. pulcherrimus* |  |
|  |  |  |  | Kunduz; Zarkharid | *An. hyrcanus* |  |
| Faulde, M. K.  (4) | 2008 | 2004 | Afghanistan | Kabul | *An. superpictus, An. fluviatilis* |  |
| Hoosh-Deghati, H.  (5) | 2017 | 2011 | Afghanistan | Herat | *An. hyrcanus* |  |
|  |  |  |  | Nangarhar; Jalalabad, Sarcha Ali khan | *An. stephensi* |  |
|  |  |  |  | Badakhshan | *An. superpictus* |  |
|  |  |  |  | Kunduz |  |  |
| Safi, N. H. Z.  (6) | 2019 | 2014-17 | Afghanistan | Kunar; Nurgal, Babur | *An. stephensi* |  |
|  |  |  |  | Laghman; Tirgari, Sawati Qarghai Laghman |  |  |
|  |  |  |  | Nangarhar; Bagrami, Saracha |  |  |
|  |  |  |  | Samarkhel; Gujranu Bella |  |  |
| Rodhain, F.  (7) | 1976 |  | Djibouti | Issa | *An. gambiae* |  |
| Carteron, B.  (8) | 1978 | 1973-76 | Djibouti | Issa | *An. gambiae* |  |
| Faulde, M. K.  (9) | 2014 | 2013 | Djibouti | Djibouti-Ambouli International Airport | *An. stephensi* |  |
| Shousha, A. T.  (10) | 1948 | 1945 | Egypt | Railways in Egypt from upper to lower | *An. gambiae* |  |
| Abdel-Malek, A. A.  (11) | 1964 | 1964 | Egypt | Cairo; Siwa | *An. sergenti* |  |
| Abdel-Malek, A. A.  (12) | 1966 | 1966 | Egypt | Cairo; Siwa | *An. sergenti* |  |
| Kamel, O. M.  (13) | 1966 | 1966 | Egypt | Dakhla Oases; Dakhla | *An. pharoensis* |  |
|  |  |  |  | Libyan Desert | *An. sergenti* |  |
|  |  |  |  | Fayum |  |  |
|  |  |  |  | Sinai Peninsula |  |  |
| Soliman, A. A.  (14) | 1967 | 1967 | Egypt | Faiyum | *An. pharoensis* |  |
| Abdel-Malek, A. A.  (15) | 1969 | 1965-67 | Egypt | Faiyum | *An. pharoensis* |  |
| Shalaby, A. M.  (16) | 1971 |  | Egypt | Alexandria | *An. pharoensis* |  |
| El‐Gayar, F. H.  (17) | 1972 | 1963 | Egypt | Nill | *An. pharoensis* |  |
| Gad, A. M.  (18) | 1984 |  | Egypt | Red Sea Governorate | *An. sergenti* |  |
| el Safi, S. H.  (19) | 1986 |  | Egypt | Faiyum Governorate; Abheet village, El-Zawya village | *An. pharoensis, An. sergenti* |  |
| Kenawy, M.  (20) | 1986 | 1982-83 | Egypt | Marsa Matrouh Governorate; Siwa Oasis | *An. sergenti, An. multicolor* |  |
|  |  |  |  | Marsa Matrouh Governorate; Cara |  |  |
| Beier, J. C.  (21) | 1987 |  | Egypt | Faiyum Governorate; Abhit Al Hagar, El-Zawya village | *An. pharoensis, An. sergenti, An. multicolor* |  |
| Gad, A. M.  (22) | 1987 | 1981-83 | Egypt | Red Sea Governorate | *An. stephensi, An. sergenti, An. multicolor* |  |
| Hilmy, N. M.  (23) | 1987 | 1983 | Egypt | Al-Qalyubia Governorate | *An. pharoensis, An. tenebrosus* |  |
| Omar, A. H.  (24) | 1987 | 1987 | Egypt | Aswan Governorate | *An. pharoensis, An. multicolor* |  |
| Zimmerman, J. H.  (25) | 1988 |  | Egypt | Imbaba; Giza Governorate, Abou Rawash | *An. tenebrosus* |  |
| Kenawy, M. A.  (26) | 1991 | 1990 | Egypt | Faiyum Governorate; Sinnuris, Tersa | *An. pharoensis, An. multicolor* |  |
| Kenawy, M. A.  (27) | 1995 |  | Egypt | Faiyum Governorate; Sinnuris, Tersa, Al Shawashna; Faiyum Governorate, El-Nazlah - Qasr El-Gebaly | *An. sergenti* |  |
| Beavers, G. M.  (28) | 1998 |  | Egypt | Matruh Governorate; Qara | *An. multicolor, An. sergenti* |  |
| Bassiouny, H. K.  (29) | 1999 | 1996 | Egypt | Faiyum Governorate; Sinnuris, Kafr Fazarah | *An. sergenti, An. multicolor, An. pharoensis* |  |
| Kenawy, M. A.  (30) | 2000 |  | Egypt | Faiyum Governorate; Sinnuris, Tersa | *An. sergenti* |  |
|  |  |  |  | Marsa Matrouh Governorate; Siwa Oasis |  |  |
| Hassan, A. N.  (31) | 2004 |  | Egypt | Beheira Governorate; Wadi El Natrun | *An. multicolor* |  |
| Abdel-Hamid, Y. M.  (32) | 2011 | 2009-10 | Egypt | Ismailia | *An. tenebrosus, An. multicolor* |  |
|  |  |  |  | Bayt El Talleh Restaurant | *An. pharoensis* |  |
|  |  |  |  | Fayed | *An. multicolor* |  |
| El-Bahnasawy, M. M.  (33) | 2011 |  | Egypt | Toshka Al Gadida | *An. sergenti, An. multicolor, An. algeriensis* |  |
| Ammar, S. E.  (34) | 2012 | 2011 | Egypt | Cairo Governorate; El Mokattam, Abusir | *An. multicolor* |  |
| Bahgat, I. M.  (35) | 2013 |  | Egypt | Ismailia Governorate | *An. multicolor, An. pharoensis* |  |
| Dahesh, S. M.  (36) | 2015 | 2014 | Egypt | Faiyum; al-Nazla | *An. sergenti, An. multicolor* |  |
| Mahmoud, D. M.  (37) | 2019 | 2015-16 | Egypt | Sabeih; Hihya, Al-Sharqia Governorate, Al Idwah | *An. multicolor, An. sergenti, An. pharoensis* |  |
| El Ela, A. A. A.  (38) | 2024 | 2020 | Egypt | Faiyum Governorate; Sinnuris | *An. multicolor* |  |
|  |  |  |  | Faiyum Governorate; Youssef Al Seddik | *An. multicolor, An. sergenti* |  |
|  |  |  |  | Faiyum Governorate; Faiyum, Tamiya | *An. multicolor* |  |
| Mohamed, H. A.  (39) | 2024 | 2019-2020 | Egypt | Beni Suef Governorate | *An. coustani, An. multicolor* |  |
| Garrett-Jones, C.  (40) | 1951 | 1950 | Iran | Chalus; Kalardasht Valley | *An. claviger, An. maculipennis, An. plumbeus, An. hyrcanus* |  |
| De Zulueta, J.  (41) | 1957 | 1956 | Iran | Isfahan; Julfa, river | *An. maculipennis* |  |
| De Zulueta, J.  (42) | 1959 | 1956 | Iran | Kazerun; Dadin Bala, Pol Abgineh | *An. sacharovi* |  |
| Golestani, J.  (43) | 1970 | 1966 | Iran | Fars; Kazerun, Shapur River, Jereh | *An. stephensi* |  |
| Motabar, M.  (44) | 1971 | 1965-67 | Iran | Fars; Kazerou, Jereh | *An. stephensi* |  |
| Manoochehri, A.  (45) | 1972 | 1965 | Iran | Hormozgan; Bandar Abbas, Gishan, Gohreh | *An. dthali* |  |
| Eshghy, N.  (46) | 1973 | 1972 | Iran | Fars; Mamasani | *An. stephensi* |  |
| Zaini, A.  (47) | 1973 | 1973 | Iran | Sistan and Baluchestan; Iranshahr, Saeid Abad, Khayer Abad | *An. culicifacies* |  |
|  |  |  |  | Sistan and Baluchestan, Hit, Chabahar |  |  |
| Javadian, E.  (48) | 1974 | 1965-68 | Iran | Khuzestan; Abadan | *An. stephensi* |  |
| Manouchehri, A. V.  (49) | 1974 | 1953-73 | Iran | Khuzestan; Izeh | *An. stephensi, An. sacharovi* |  |
|  |  |  |  | Ardabil; Meshginshahr | *An. sacharovi* |  |
| Sahabi, Z.  (50) | 1974 |  | Iran | Fars; Kazerun | *An. stephensi* |  |
| Manouchehri, A. V.  (51) | 1976 | 1957 | Iran | Khuzestan; Behbahan | *An. fluviatilis* |  |
|  |  | 1959 |  | Fars; Jahrom, Kazeroun |  |  |
|  |  |  |  | Kerman; Jiroft |  |  |
|  |  | 1960 |  | Hormozgan; Bandar Abbas |  |  |
| Zaim, M.  (52) | 1986 |  | Iran | Hgrmozgan; Chelow | *An. stephensi, An. fluviatilis* |  |
| Manouchehri, A. V.  (53) | 1988 | 1976-86 | Iran | Hormozgan; Minab | *An. stephensi* |  |
| Weiser, J.  (54) | 1991 | 1988 | Iran | Gilan; Lahijan | *An. maculipennis* |  |
| Zaim, M.  (55) | 1991 | 1987 | Iran | Sistan and Baluchestan; Sarbaz, Rask, Iranshah, Ghasre Ghand, Chabahar, Nikshahr | *An. culicifacies* |  |
| Zaim, M.  (56) | 1992 | 1990-91 | Iran | Sistan and Baluchestan; Nikshahr, Zeineddini | *An. pulcherrimus* |  |
| Zaim, M.  (57) | 1993 | 1991 | Iran | Sistan and Baluchestan; Ghasre Ghand | *An. culicifacies, An. pulcherrimus* |  |
| Zaim, M.  (58) | 1995 | 1990-92 | Iran | Sistan and Baluchestan | *An. culicifacies* |  |
| Yaghoobi-Ershadi, M. R.  (59) | 2001 | 1998-99 | Iran | Ardabil; Parsabad, Garmi Angut | *An. sacharovi* |  |
| Naddaf, S. R.  (60) | 2003 | 1983-2002 | Iran | Hormozgan; Minab, Siahoo | *An. fluviatilis* |  |
|  |  |  |  | Sistan and Baluchestan; Daman |  |  |
|  |  |  |  | Kerman; Kahnuj |  |  |
|  |  |  |  | Fars; Kazerun, khesht |  |  |
|  |  |  |  | Bushehr; Dashtestan |  |  |
| Sedaghat, M. M.  (61) | 2003 | 1983-2002 | Iran | Esfahan; Fereidon Shahr, Ahmad Abad, Kheirabad, Zardanjan | *An. maculipennis* |  |
|  |  |  |  | Mazandaran; Neka, Qaem shahr, Amol |  |  |
|  |  |  |  | Tehran; Jajrud |  |  |
|  |  |  |  | Zanjan; Zanjan |  |  |
|  |  |  |  | Gilan; Masuleh, Rasht |  |  |
|  |  |  |  | Golestan; Gonbad-e Kavus, Gorgan, Ramian |  |  |
|  |  |  |  | Kohgiluyeh and Boyer-Ahmad; Yasuj |  |  |
|  |  |  |  | Mazandaran; Babol, Sari | *An. sacharovi* |  |
|  |  |  |  | Fars; Kazerun |  |  |
|  |  |  |  | Mazandaran; Behshahr | *An. maculipennis, An. sacharovi* |  |
|  |  |  |  | West Azerbaijan; Maku, Poldasht |  |  |
| Vatandoost, H.  (62) | 2004 |  | Iran | Hormozgan | *An. stephensi, An. fluviatilis, An. dthali* |  |
| Basseri, H. R.  (63) | 2005 | 2002-3 | Iran | Kerman; Kahnuj | *An. fluviatilis, An. stephensi, An. dthali, An. culicifacies, An. superpictus* |  |
| Ghavami, M. B.  (64) | 2005 | 2003 | Iran | Zanjan; Qarah Buteh | *An. maculipennis* |  |
| Vatandoost, H.  (65) | 2005 |  | Iran | Kerman; Kahnuj | *An. stephensi, An. dthali* |  |
| Davari, B.  (66) | 2006 | 2004-5 | Iran | Fars; Kazerun | *An. stephensi* |  |
|  |  |  |  | Sistan and Baluchestan Province; Iranshah |  |  |
|  |  |  |  | Hormozgan; Bandar Abbas |  |  |
| Djadid, N. D.  (67) | 2006 | 2005 | Iran | Kerman | *An. stephensi* |  |
|  |  |  |  | Fars; Kazerun |  |  |
|  |  |  |  | Sistan and Baluchestan; Nikshahr, Chabahar, Iranshahr, Khash |  |  |
|  |  |  |  | Hormozgan; bandar Abbas |  |  |
| Khoobdel, M.  (68) | 2006 | 2004 | Iran | Fars; Kazerun | *An. stephensi* |  |
| Vatandoost, H.  (69) | 2006 | 2002 | Iran | Hormozgan; Bandar Abbas | *An. stephensi* |  |
| Abai, M. R.  (70) | 2007 | 2004-5 | Iran | East Azerbaijan; Kaleibar | *An. claviger, An. hyrcanus, An. pseudopictus, An. superpictus, An. sacharovi* |  |
|  |  |  |  | East Azerbaijan; Ahar, Kaleibar, Tabriz | *An. maculipennis* |  |
|  |  |  |  | East Azerbaijan; Tabriz | *An. superpictus* |  |
| Djadid, N. D.  (71) | 2007 | 2006 | Iran | Sistan and Baluchestan; Saravan, Iranshahr, Nikshahr, Khash, Chabahar | *An. culicifacies* |  |
| Djadid, N. D.  (72) | 2007 | 1997-8-9-2001 | Iran | East Azerbayjan; Ardebil, Guilan, Mazandaran, Khorassan | *An. maculipennis* |  |
| Moosa-Kazemi, S. H.  (73) | 2007 | 2005 | Iran | Sistan and Baluchestan; Chabahar | *An. culicifacies, An. stephensi, An. pulcherrimus, An. dthali, An. fluviatilis* |  |
| Oshaghi, M. A.  (74) | 2007 |  | Iran | Ardebil; Lorestan; Zanjan; Qom; Razavi; Khorasan; Fars; Kerman; Sistan and Baluchestan | *An. superpictus* |  |
| Vatandoost, H.  (75) | 2007 | 2002 | Iran | Hormozgan; Bandar Abbas | *An. dthali* |  |
| Abai, M. R.  (76) | 2008 | 2006 | Iran | Kerman; Jiroft, Daryacheh, Sephidbaz, Saghari, Dow Sari | *An. stephensi* |  |
| Basseri, H. R.  (77) | 2008 | 2005 | Iran | Sistan and Baluchestan Province; Iranshahr | *An. stephensi* |  |
| Dinparast Djadid, N.  (78) | 2008 | 2005 | Iran | Fars; Kazeroon | *An. stephensi* |  |
|  |  |  |  | Sistan and Baluchestan; Saravan, Chabahar, Nikshahr |  |  |
| Ghavami, M. B.  (79) | 2008 |  | Iran | Zanjan; Zanjan | *An. maculipennis* |  |
| Rasoolian, M.  (80) | 2008 | 2007 | Iran | Kazerun; Islam-abad, Ali-abad Ghuri, Ghaemieh, Pirsabz | *An. dthali* |  |
|  |  |  |  | Kazerun; Pirsabz | *An. fluviatilis* |  |
|  |  |  |  | Kazerun; Arabe chegini | *An. superpictus* |  |
|  |  |  |  | Kazerun; Garm-abad, Fath-abad | *An. sacharovi* |  |
| Shemshad, K.  (81) | 2008 | 2003 | Iran | Sistan and Baluchestan; Sarbaz | *An. superpictus* |  |
|  |  |  |  | Ardabil; Garmi Angut |  |  |
|  |  |  |  | Lorestan; Azna |  |  |
| Djadid, N. D.  (82) | 2009 | 2003 | Iran | Ardabil; Parsabad | *An. hyrcanus* |  |
|  |  | 2005 |  | Gilan; Amlash, Astaneh-AshraÞeh, Fuman, Rezvanshahr, Talesh, Rudsar, Astara, Langarud |  |  |
|  |  | 2007 |  | Khuzestan; Shadegan |  |  |
| Azari-Hamidian, S.  (83) | 2010 | 2005-7 | Iran | Hormozgan; Bandar Abbas | *An. stephensi* |  |
|  |  |  |  | Hormozgan; Qeshm | *An. culicifacies, An. dthali, An. fluviatilis, An. stephensi, An. turkhudi* |  |
|  |  |  |  | Hormozgan; Abumusa | *An. fluviatilis* |  |
| Basseri, H.  (84) | 2010 | 2007-8 | Iran | Hormozgan; Bandar Abbas, Sistan and Baluchestan | *An. stephensi, An. culicifacies, An. fluviatilis, An. dthali* |  |
| Farzinnia, B.  (85) | 2010 | 2008 | Iran | Qom; Qahan, Aghelak, Rahjerd-e Sharqi, Ahmadabad, Emamzadeh Esmail | *An. superpictus, An. claviger* |  |
| Hasasan, V.  (86) | 2010 | 1998-99 | Iran | Gilan; Astara | *An. maculipennis* |  |
| Hassan, V.  (87) | 2010 | 2008 | Iran | Kerman; Kahnuj | *An. stephensi, An. dthali, An. superpictus, An. culicifacies, An. fluviatilis* |  |
| Nikookar, Sh  (88) | 2010 | 2009 | Iran | Mazandaran; Neka | *An. plumbeus* |  |
| Raeisi, A.  (89) | 2010 | 2006 | Iran | Sistan and Baluchestan; Iranshahr, Malekabad | *An. stephensi* |  |
| Shahandeh, Kh  (90) | 2010 | 2006 | Iran | Hormozgan; Siahoo | *An. fluviatilis, An. stephensi, An. dthali* |  |
| Ahmad, M.  (91) | 2011 | 2006-7 | Iran | Kerman; Jiroft | *An. culicifacies, An. stephensi, An. superpictus, An. fluviatilis, An. sergenti, An. pulcherrimus, An. turkhudi, An. dthali* |  |
| Azari-Hamidian, S.  (92) | 2011 | 2000 | Iran | Gilan | *An. claviger, An. hyrcanus, An. maculipennis, An. Plumbeus, An. superpictus* |  |
| Azari-Hamidian, S.  (93) | 2011 | 2005-9 | Iran | North Khorasan | *An. claviger, An. maculipennis, An. pulcherrimus, An. superpictus* |  |
| Hassan, V.  (94) | 2011 | 2005-7 | Iran | Hormozgan; Minab | *An. stephensi, An. dthali, An. superpictus, An. fluviatilis, An. multicolor, An. pulcherrimus, An. turkhudi* |  |
| Mehravaran, A.  (95) | 2011 | 2007-8 | Iran | Kerman; Jiroft | *An. fluviatilis* |  |
|  |  |  |  | Sistan and Baluchestan; Chabahar |  |  |
| Oshaghi, M. A.  (96) | 2011 | 2008 | Iran | Ardabil; Parsabad | *An. maculipennis, An. sacharovi* |  |
| Vatandoost, H.  (97) | 2011 | 2005 | Iran | Sistan and Baluchestan | *An. culicifacies* |  |
| Basseri, H. R.  (98) | 2012 | 2007-8 | Iran | Sistan and Baluchestan; Sarbaz | *An. culicifacies, An. fluviatilis, An. stephensi* |  |
| Hanafi-Bojd, A. A.  (99) | 2012 | 2009-10 | Iran | Hormozgan; Bashagard | *An. culicifacies, An. dthali, An. stephensi, An. superpictus, An. fluviatilis, An. turkhudi, An. moghulensis, An. apoci* |  |
| Khoobdel, M.  (100) | 2012 | 2009-10 | Iran | Hormozgan; Qeshm, Kish Island | *An. stephensi* |  |
| Mehravaran, A.  (101) | 2012 |  | Iran | Kerman; Jiroft | *An. stephensi* |  |
| Saghafipour, A.  (102) | 2012 | 2008-9 | Iran | Qom; Aghelak, Ahmadabad, Dastjerd, Ghahan, QalehCham | *An. claviger* |  |
|  |  |  |  | Qom; Rahjerd | *An. marteri* |  |
|  |  |  |  | Qom; Dastjerd, Emamzadeh Esmaeil | *An. superpictus* |  |
|  |  |  |  | Qom; Dastjerd | *An. turkhudi* |  |
| Vatandoost, H.  (103) | 2012 |  | Iran | East Azerbaijan; Larijan, Guneh Qarshu, Jafar Abad, Mahmoudābād | *An. sacharovi* |  |
| Vatandoost, H.  (104) | 2012 | 2011 | Iran | Sistan and Baluchestan; Chabahar | *An. stephensi* |  |
| Banafshi, O.  (105) | 2013 | 2005-6 | Iran | Kurdistan; Baneh, Bijar, Sanandaj, Sarvabad | *An. claviger* |  |
|  |  |  |  | Kurdistan; Baneh, Bijar, Dehgolan, Divandarreh, Kamyaran, Sanandaj, Saqqez, Sarvabad | *An. maculipennis, An. superpictus* |  |
| Nejati, J.  (106) | 2013 | 2012 | Iran | Sistan and Baluchestan; Sarbaz | *An. superpictus* |  |
| Shahi, M.  (107) | 2013 | 2009 | Iran | hormozgan; Bashagard, Siahoo | *An. stephensi* |  |
| Soleimani-Ahmadi, M.  (108) | 2013 | 2010-11 | Iran | Hormozgan; Dehbārez | *An. dthali, An. stephensi, An. culicifacies, An. superpictus, An. turkhudi, An. apoci* |  |
| Soltani, A.  (109) | 2013 | 2011 | Iran | Hormozgan; Bandar Abbas port, Minab | *An. stephensi* |  |
|  |  |  |  | Sistan and Baluchestan; Chabahar Sea Port, Iranshahr, Bampur, Abtar, Sarbaz |  |  |
| Amani, H.  (110) | 2014 | 1997 | Iran | Lorestan; Aligudarz, Eastern Zalaghi | *An. superpictus, An. maculipennis, An. turkhudi, An. dthali, An. claviger* |  |
|  |  |  |  | Lorestan; Aligudarz, Eastern Zez | *An. marteri, An. superpictus, An. maculipennis, An. turkhudi, An. dthali, An. claviger, An. marteri* |  |
|  |  |  |  | Lorestan; Aligudarz, Farsesh | *An. superpictus, An. maculipennis, An. turkhudi, An. dthali, An. claviger* |  |
|  |  |  |  | Lorestan; Aligudarz, Mahroo | *An. superpictus, An. turkhudi, An. dthali* |  |
|  |  |  |  | Lorestan; Aligudarz, Eastern Berberud | *An. superpictus, An. maculipennis, An. claviger* |  |
|  |  |  |  | Lorestan; Aligudarz, Eastern Pachehlak | *An. superpictus, An. maculipennis* |  |
| Chavshin, A. R.  (111) | 2014 |  | Iran | Sistan and Baluchestan; Iranshahr, Anguri district, Saraydan | *An. culicifacies* |  |
| Khoshdel-Nezamiha, F.  (112) | 2014 | 2012 | Iran | West Azerbaijan Province | *An. claviger, An. maculipennis, An. superpictus* |  |
| Zahirnia, A. H.  (113) | 2014 | 2013 | Iran | Hamadan; Salehabad, Varkaneh | *An. superpictus, An. maculipennis* |  |
|  |  |  |  | Hamadan; Salehabad | *An. stephensi* |  |
| Ataie, A.  (114) | 2015 | 2011 | Iran | East Azerbaijan; Ahar | *An. maculipennis, An. sacharovi, An. claviger* |  |
| Chavshin, A. R.  (115) | 2015 | 2013 | Iran | West Azerbaijan Province | *An. maculipennis* |  |
| Farhadinejad, R.  (116) | 2015 | 2012 | Iran | Khuzestan; Bandar-e Mahshahr | *An. stephensi, An. superpictus, An. dthali, An. pulcherrimus* |  |
| Fathian, M.  (117) | 2015 | 2010 | Iran | Sistan and Baluchestan; Chabahar | *An. stephensi* |  |
| Ladonni, H.  (118) | 2015 | 2003-6 | Iran | Isfahan Province; Fereydunshahr | *An. dthali, An. turkhudi* |  |
|  |  |  |  | Isfahan; Isfahan, Tiran, Chadegan, Khomeinishahr, Fereydunshahr, Falavarjan, Lenjan, Mobarakeh, Najafabad | *An. maculipennis* |  |
|  |  |  |  | Isfahan; Chadegan, Fereydunshahr | *An. marteri* |  |
|  |  |  |  | Isfahan; Nain | *An. multicolor* |  |
|  |  |  |  | Isfahan; Ardestan, Tiran, Chadegan, Semirom, Shahinshahr, Shahreza, Faridan, Fereydunshahr, Golpayegan, Mobarakeh, Nain | *An. superpictus* |  |
| Maghsoodi, N.  (119) | 2015 | 2011-12 | Iran | Khuzestan; Izeh | *An. stephensi, An. dthali, An. superpictus, An. fluviatilis* |  |
| Moosa-Kazemi, S. H.  (120) | 2015 | 2012 | Iran | Kurdistan; Hassan Abad, Hoseynabad-e Jonubi, | *An. maculipennis, An. superpictus* |  |
|  |  |  |  | Kermanshah | *An. maculipennis, An. superpictus, An. turkhudi* |  |
| Nikookar, S. H.  (121) | 2015 | 2009 | Iran | Mazandaran; Neka | *An. claviger, An. maculipennis, An. plumbeus, An. superpictus* |  |
| Soleimani-Ahmadi, M.  (122) | 2015 | 2013 | Iran | Hormozgan; Minab | *An. moghulensis, An. stephensi, An. dthali, An. culicifacies, An. fluviatilis, An. superpictus, An. turkhudi, An. pulcherrimus, An. multicolor, An. apoci* |  |
| Abai, M. R.  (123) | 2016 | 2014 | Iran | Fars; Kazerun | *An. stephensi* |  |
| Gorouhi, M. A.  (124) | 2016 | 2013 | Iran | Sistan and Baluchestan; Chabahar | *An. stephensi* |  |
| Khoshdel-Nezamiha, F.  (125) | 2016 | 2012 | Iran | West Azerbaijan; Berisu, Bavan, Yarim Qayeh | *An. maculipennis* |  |
|  |  |  |  | West Azerbaijan; Mahabad, Beran | *An. superpictus* |  |
| Nikookar, S. H.  (126) | 2016 | 2014 | Iran | Mazandaran | *An. claviger, An. hyrcanus, An. maculipennis, An. marteri, An. plumbeus, An. pseudopictus* |  |
| Omrani, S. M.  (127) | 2016 | 2012 | Iran | Chaharmahal and Bakhtiari; Kiar | *An. superpictus* |  |
| Sofizadeh, A.  (128) | 2016 | 2012-13 | Iran | Golestan Province; Kalaleh | *An. maculipennis, An. superpictus* |  |
| Soltani, Z.  (129) | 2016 | 2012 | Iran | Fars; Farashband | *An. superpictus, An. dthali* |  |
| Yeryan, M.  (130) | 2016 | 2012 | Iran | Hormozgan; Bandar-e-Jask | *An. stephensi, An. culicifacies, An. fluviatilis, An. dthali, An. moghulensis, An. turkhudi* |  |
| Hoosh-Deghati, H.  (5) | 2017 | 2011 | Iran | Sistan and Baluchestan; Chabahar | *An. stephensi, An. fluviatilis, An. culicifacies, An. sergenti* |  |
| Keshavarzi, D.  (131) | 2017 | 2012 | Iran | Fars; Khonj, Mohr, Darab, Zarrin Dasht, Lamerd, Larestan | *An. dthali* |  |
|  |  |  |  | Fars; Khonj, Mohr, Darab, Lamerd | *An. stephensi* |  |
|  |  |  |  | Fars; Mohr | *An. fluviatilis, An. superpictus* |  |
|  |  |  |  | Fars; Larestan | *An. superpictus* |  |
| Nikookar, S. H.  (132) | 2017 | 2014 | Iran | Mazandaran | *An. maculipennis, An. marteri* |  |
| Omrani, S. M.  (133) | 2017 | 2013 | Iran | Chaharmahal and Bakhtiari; Kiar | *An. superpictus* |  |
| Soltani, Z.  (134) | 2017 | 2012 | Iran | Fars; Firouzabad, Zanjiran, Dehrud, Dadenjan | *An. superpictus* |  |
|  |  |  |  | Fars; Firouzabad, Mehkuyeh, Zanjiran, Dehrud, Dadenjan, Banaf | *An. dthali* |  |
| Yazdi, F.  (135) | 2017 | 2012-13 | Iran | Mazandaran; Nur | *An. hyrcanus, An. maculipennis* |  |
| Azari-Hamidian, S.  (136) | 2018 | 2015-16 | Iran | Gilan; Rudbar | *An. claviger* |  |
|  |  |  |  | Gilan; Rasht, Rudsar | *An. hyrcanus* |  |
|  |  |  |  | Gilan; Rudsar, Rasht, Bandar Anzali, Rudsar, Hashtpar, Siahkal | *An. maculipennis* |  |
| Gorouhi, M. A.  (137) | 2018 | 2015 | Iran | Sistan and Baluchestan; Chabahar | *An. stephensi* |  |
| Nejati, J.  (138) | 2018 | 2015 | Iran | Sistan and Baluchestan; Sarbaz | *An. subpictus* |  |
| Nikookar, S. H.  (139) | 2018 | 2014 | Iran | Mazandaran; Behshahr, Juybar, Noshahr | *An. claviger* |  |
|  |  |  |  | Mazandaran; Behshahr, Neka, Sari, Ghaemshahr, Amol, Mahmudabad, Nur, Noshahr, Ramsar | *An. hyrcanus* |  |
|  |  |  |  | Mazandaran; Galugah, Behshahr, Neka, Sari, Ghaemshahr, Savadkuh, Juybar, Amol, Nur, Noshahr, Chalus, Tonekabon, Ramsar | *An. maculipennis, An. pseudopictus* |  |
|  |  |  |  | Mazandaran; Noshahr, Behshahr, Sari, Juybar, Chalus, Tonekabon, Ramsar | *An. marteri* |  |
|  |  |  |  | Mazandaran; Galugah, Behshahr | *An. superpictus* |  |
| Sofizadeh, A.  (140) | 2018 | 2015 | Iran | Golestan; Minoodasht | *An. hyrcanus, An. pseudopictus* |  |
|  |  |  |  | Golestan; Kalaleh, Galikesh | *An. maculipennis* |  |
|  |  |  |  | Golestan; Kalaleh | *An. superpictus* |  |
| Asadi Saatlou, Z.  (141) | 2019 | 2015-18 | Iran | West Azerbaijan; Urmia, Nazlu, Jarchelu, Kuran | *An. maculipennis* |  |
|  |  |  |  | East Azerbaijan; Khodaafarin, Larijan |  |  |
|  |  |  |  | Zanjan, Qarah Buteh |  |  |
|  |  |  |  | Gilan; Langrood, Astara, Masal, Talesh, Siahkal |  |  |
| Gholami, S.  (142) | 2019 | 2015-16 | Iran | West Azerbaijan; Poldasht, Shahindej, Oshnavieh, Maku | *An. maculipennis, An. sacharovi, An. claviger, An. superpictus* |  |
|  |  |  |  | Ardabil; Parsabad-Moghan, Aslan-Duz, Meshginshahr | *An. maculipennis, An. sacharovi, An. claviger, An. hyrcanus* |  |
|  |  |  |  | East Azerbaijan; Kalibar, Azarshahr |  |  |
|  |  |  |  | North Khorasan; Bojnord, Shirvan | *An. maculipennis, An. claviger, An. hyrcanus, An. superpictus* |  |
|  |  |  |  | Golestan; Gorgan |  |  |
|  |  |  |  | Golestan; Gorgan | *An. pseudopictus* |  |
| Karami, M.  (143) | 2019 | 2015 | Iran | Mazandaran; Amol | *An. maculipennis, An. pseudopictus, An. hyrcanus* |  |
| Paksa, A.  (144) | 2019 | 2017 | Iran | East Azerbaijan Province, Ahar | *An. superpictus* |  |
|  |  |  |  | East Azerbaijan; Qareh Tappeh, Zināb | *An. maculipennis, An. hyrcanus* |  |
|  |  |  |  | East Azerbaijan; Zināb | *An. hyrcanus* |  |
| Edalat, H.  (145) | 2020 | 2015 | Iran | Kerman; Qaleh Ganj, Rameshk | *An. stephensi, An. culicifacies, An. superpictus, An. dthali, An. fluviatilis* |  |
| Ghavami, M. B.  (146) | 2020 | 2016 | Iran | Zanjan; Gheytoul, Ghara Bouta, Moshampa, Kheyrabad, Sari Aghol | *An. maculipennis* |  |
| Khoobdel, M.  (147) | 2020 | 2017 | Iran | Bushehr; Bushehr | *An. stephensi, An. superpictus, An. dthali, An. subpictus* |  |
| Mojahedi, A. R.  (148) | 2020 | 2014-15 | Iran | Hormozgan; Siahoo | *An. dthali, An. fluviatilis, An. culicifacies, An. stephensi* |  |
| Asgarian, T. S.  (149) | 2021 | 2019 | Iran | Isfahan; Kashan | *An. maculipennis, An. superpictus* |  |
| Faraji-Fard, P.  (150) | 2021 | 2015 | Iran | Khuzestan; Shoushtar, Nur Ali | *An. stephensi, An. pulcherrimus* |  |
| Kassiri, H.  (151) | 2021 |  | Iran | Isfahan; Lenjan, Mobarakeh | *An. maculipennis* |  |
| Mirahmadi, H.  (152) | 2021 | 2018 | Iran | Sistan and Baluchestan; Chabahar, Konarak | *An. stephensi, An. culicifacies, An. dthali, An. polacrimus* |  |
| Moosa-Kazemi, S. H.  (153) | 2021 | 2019 | Iran | Isfahan; Kashan, Qamsar, Barzok | *An. superpictus* |  |
|  |  |  |  | Isfahan; Qamsar, Niyasar, Barzok | *An. maculipennis* |  |
|  |  |  |  | Isfahan; Niyasar | *An. claviger* |  |
|  |  |  |  | Isfahan; Barzok | *An. turkhudi* |  |
| Moosa-Kazemi, S. H.  (154) | 2021 | 2017 | Iran | Lorestan; Khorramabad | *An. dthali, An. superpictus, An. sacharovi, An. maculipennis* |  |
|  |  |  |  | Lorestan; Dowreh | *An. dthali, An. superpictus, An. stephensi, An. sacharovi, An. maculipennis* |  |
|  |  |  |  | Lorestan; Selseleh | *An. maculipennis, An. dthali* |  |
| Soltani, A.  (155) | 2021 | 2017-18 | Iran | Fars; Qir-o-Karzin | *An. stephensi, An. dthali, An. superpictus* |  |
| Sharifi, F.  (156) | 2022 | 2019 | Iran | Kurdistan; Baneh, Marivan, Kamyaran, Divandarreh | *An. claviger* |  |
|  |  |  |  | Kurdistan; Sanandaj, Baneh, Marivan, Sarvabad, Kamyaran, Divandarreh | *An. maculipennis, An. superpictus* |  |
| Amirkafi, A.  (157) | 2023 | 2018 | Iran | Kerman; Qaleh Ganj, Rameshk | *An. stephensi, An. culicifacies, An. superpictus, An. dthali, An. fluviatilis, An. moghulensis, An. turkhudi* |  |
| Hajibeygi, R.  (158) | 2023 | 2022 | Iran | Sistan and Baluchestan; Saravan | *An. stephensi* |  |
| Nasiri, Z.  (159) | 2023 | 2021 | Iran | Bushehr; Dashtestan | *An. superpictus, An. stephensi, An. dthali, An. fluviatilis* |  |
| Askari, F.  (160) | 2024 | 2023 | Iran | Golestan; Ramiyan, Kordkuy, Ali Abad, Gonbad-e Kavus, Bandar Gaz, Torkaman, Galikesh, Maraveh Tappeh, Kalaleh | *An. hyrcanus* |  |
| Etherington, D.  (161) | 1946 | 1945 | Iraq | Basreh; Shatt-el-Arab | *An. sacharovi* |  |
|  |  |  |  | Sulaimaniya | *An. sacharovi, An. superpictus* |  |
| Pringle, G.  (162) | 1952 |  | Iraq | Diyala Liwa | *An. superpictus, An. stephensi* |  |
| Gramiccia, G.  (163) | 1958 | 1957 | Iraq | Basra; Om-el-Resas | *An. stephensi* |  |
| Abul-Hab, J.  (164) | 1969 | 1955 | Iraq | Nineveh, Kurdistan Region, Duhok | *An. maculipennis, An. sacharovi, An. superpictus* |  |
| Niazi, A.  (165) | 1973 | 1965-71 | Iraq | Basrah; Basrah | *An. stephensi* |  |
| Al-Ghoury, A. A.  (166) | 2005 | 2002 | Iraq | Babylon Governorate; Hillah | *An. stephensi, An. pulcherrimus* |  |
| Hantosh, H. A.  (167) | 2012 | 2009 | Iraq | Al-Qādisiyyah Governorate; Najaf Governorate; Najaf, Al Anbar Governorate, Babylon Governorate, Basrah, Diyala Governorate, Karbala, Maysan Governorate, Dhi Qar, Wasit Governorate | *An. pulcherrimus* |  |
|  |  |  |  | Najaf Governorate, Najaf, Babylon Governorate, Basrah, Karbala, Maysan Governorate, Dhi Qar Governorate, Wasit Governorate, | *An. stephensi* |  |
|  |  |  |  | Diyala Governorate, Nineveh Governorate, Saladin Governorate | *An. superpictus* |  |
|  |  |  |  | Nineveh Governorate, Saladin Governorate | *An. sacharovi* |  |
| Farid, M. A.  (168) | 1954 | 1951 | Jordan | Jordan Valley | *An. sergenti, An. superpictus, An. sacharovi, An. hyrcanus, An. multicolor, An. pharoensis* |  |
|  |  |  |  | North Shuneh village | *An. pharoensis* |  |
| Amr, Z. S.  (169) | 1996 | 1997 | Jordan | Jarash Governorate; Al-Mashreh | *An. algeriensis* |  |
|  |  |  |  | Jarash Governorate; Dayr al Liyat, Al-Mashreh, Irbid Governorate; Ain Al-Tlab | *An. claviger* |  |
|  |  |  |  | Ajloun Governorate; Wadi Rajib | *An. marteri* |  |
|  |  |  |  | Irbid Governorate; Ain Al-Moalaka | *An. sacharovi* |  |
|  |  |  |  | Sweimeh | *An. dthali* |  |
|  |  |  |  | Jarash Governorate; Madi area | *An. multicolor* |  |
|  |  |  |  | Jarash Governorate; Dayr al Liyat | *An. sergenti* |  |
|  |  |  |  | Irbid Governorate; Ain Al-Moalaka, Jarash Governorate; Tabaqat Faha | *An. superpictus* |  |
| Cropper, J.  (170) | 1902 | 1900 | Lebanon | Beyrout; Beyrout river, sidon, Ayn ad Dulb | *An. superpictus* |  |
|  |  |  |  | Tyre, Ras El Ain, Plain of Acca, Ras an-Nakura | *An. maculipennis* |  |
|  |  |  |  | Plain of Acca, Ras an-Nakura | *An. superpictus* |  |
| Garrett-Jones, C.  (171) | 1954 | 1951 | Lebanon | Bouqaia, Orontes River | *An. sacharovi* |  |
| Knio, K. M.  (172) | 2005 | 1999-2001 | Lebanon | Ain El Roumaheh, Sabtieh, Zalka, Nabay, Rabweh | *An. claviger* |  |
| Shalaby, A. M.  (16) | 1971 |  | Libya | Tripoli | *An. multicolor* |  |
| Bailly Choumara, H.  (173) | 1973 | 1968 | Moroco | Larache; Al-Araish | *An. labranchiae* |  |
| Deschiens, R.  (174) | 1975 | 1974 | Moroco | Al Hassan ADDakhil dam | *An. sergenti, An. multicolor, An. hispaniola* |  |
| Faraj, C.  (175) | 2004 | 2002 | Moroco | Khouribga, Taounate, Al Hoceima, Chefchaouen, Fes, Khemisset, El Kelaa des Sraghna, Ben Slimane | *An. maculipennis* |  |
| Faraj, C.  (176) | 2008 | 2005 | Moroco | Knitra, Khouribga, Larache, Salé | *An. labranchiae* |  |
| Faraj, C.  (177) | 2008 |  | Moroco | Chefchaouen | *An. dthali* |  |
| Faraj, C.  (178) | 2009 | 2005 | Moroco | Chefchaouen; Assoul | *An. sergenti, An. cinereus, An. dthali, An. labranchiae, An. claviger* |  |
|  |  |  |  | Chefchaouen; Mizgane | *An. sergenti, An. cinereus, An. dthali, An. labranchiae, An. claviger* |  |
| Majda, L.  (179) | 2010 | 2009 | Moroco | Larache; Laouamra, Zouada | *An. labranchiae* |  |
|  |  |  |  | Larache; Rissana Janoubia, Rissana Chamalia, Ayacha, Bni Arouss, Zaaroura, Souk L'Qolla, Souk Tolba | *An. labranchiae, An. cinereus* |  |
| Adlaoui, E.  (180) | 2011 | 2008 | Moroco | Oued Loukos | *An. labranchiae* |  |
| Laboudi, M.  (181) | 2011 | 2007-8 | Moroco | Bab Berrad, Chefchaouen, Rommani, Beggara, Laouamra | *An. labranchiae* |  |
| Laboudi, M.  (182) | 2012 | 2009 | Moroco | Larache; Laouamra, Zouada | *An. labranchiae* |  |
|  |  |  |  | Larache; Rissana Janoubia, Rissana Chamalia, Ayacha, Bni Arouss, Zaaroura, Souk L'Qolla, Souk Tolba | *An. labranchiae, An. cinereus* |  |
| El-Akhal, F.  (183) | 2016 | 2014 | Moroco | Fes; Fes | *An. labranchiae* |  |
| Filali Mouatassem, T.  (184) | 2019 | 2015-16 | Moroco | Fes; Fes | *An. maculipennis, An. sergenti, An. cinereus* |  |
| Mouatassem, T. F.  (185) | 2021 | 2015-16 | Moroco | Fes; Fes | *An. maculipennis, An. sergenti* |  |
| Filali Mouatassem, T.  (186) | 2024 | 2015-16 | Moroco | Fes; Fes | *An. maculipennis, An. sergenti, An. cinereus* |  |
| Roberts, D.  (187) | 1996 |  | Oman | Al Bāţinah, Barka city | *An. stephensi, An. culicifacies* |  |
| Baomar, A. T.  (188) | 2000 | 1998 | Oman | Dhofar Governorate | *An. dthali, An. sergenti, An. stephensi* |  |
| Carmichael, G. T.  (189) | 1972 |  | Pakistan | Karachi | *An. stephensi* |  |
| Akiyama, J.  (190) | 1974 | 1962 | Pakistan | Punjab; Lahore, Rakh Dala | *An. culicifacies, An. stephensi* |  |
| Reisen, W. K.  (191) | 1976 | 1975 | Pakistan | Punjab; Lahore, Sattoki | *An. culicifacies* |  |
| Reisen, W. K.  (192) | 1978 | 1976-77 | Pakistan | Punjab; Lahore | *An. culicifacies, An. stephensi* |  |
| Reisen, W. K.  (193) | 1978 | 1976 | Pakistan | Punjab; Lahore, Sattoki | *An. pulcherrimus, An. culicifacies, An. stephensi, An. Annularis, An. nigerrimus, An. subpictus* |  |
| Reisen, W. K.  (194) | 1979 | 1978 | Pakistan | Punjab; Lahore, Khano-Harni | *An. subpictus* |  |
| Aslamkhan, M.  (195) | 1981 |  | Pakistan | Punjab; Lahore, | *An. stephensi* |  |
| Hemingway, J.  (196) | 1982 | 1947 | Pakistan | Punjab; Lahore, | *An. stephensi* |  |
| Reisen, W. K.  (197) | 1982 | 1979-80 | Pakistan | Punjab; Kasur, Kot Baghicha | *An. culicifacies* |  |
| Reisen, W. K.  (198) | 1982 | 1978 | Pakistan | Punjab; Lahore, Khano-Harni | *An. subpictus, An. culicifacies, An. stephensi* |  |
| Mahmood, F.  (199) | 1984 | 1979-83 | Pakistan | Punjab; Lahore, Kasur, Sindh, Karachi City | *An. stephensi* |  |
| Mahmood, F.  (200) | 1984 | 1979-83 | Pakistan | Punjab; Lahore, Khan Harni, Green Town, Sadoki, Khizarabad, Singh Wala, Khara, Shahzada, Khairpur Malian, Jaffarabad, Sohbatpur, Rajanpur, Dera Ghazi Khan, Kasur, Punjab, Kot Baghicha, Sattoki, Kot Manan, Muzaffargarh, Sialkot, Karnwali , Dalhozi, Antowali, Saharan, Baghyana Kalan, Khanke MorrKhanke Morr | *An. culicifacies* |  |
| Nalin, D. R.  (201) | 1985 |  | Pakistan | Karachi City, Sindh, Karachi | *An. stephensi* |  |
| Hewitt, S.  (202) | 1994 |  | Pakistan | Azhakhel Afghan Refugee Camp | *An. aunnuaris, An. culicifacies, An. fluviatilis, An. nigerrimus, An. pulcherrimus, An. stephensi, An. subpictus* |  |
| Hewitt, S.  (203) | 1995 |  | Pakistan | Khyber Pakhtunkhwa | *An. stephensi, An. subpictus* |  |
| Bouma, M. J.  (204) | 1996 | 1989 | Pakistan | South Waziristan Agency | *An. culicifacies, An. stephensi* |  |
| Rowland, M.  (205) | 2001 |  | Pakistan | frontier province, Hangu valley | *An. stephensi, An. culicifacies, An. subpictus, An. fluviatilis, An. annularis* |  |
| Graham, K.  (206) | 2002 | 1999 | Pakistan | Azhakhel Afghan Refugee Camp | *An. nigerrimus, An. pulcherrimus, An. stephensi* |  |
| Mukhtar, M.  (207) | 2003 | 2000 | Pakistan | Bahawalnagar, Punjab, Haroonabad | *An. stephensi, An. subpictus, An. culicifacies, An. pulcherrimus* |  |
| Yasinzai, M. I.  (208) | 2004 | 2002 | Pakistan | Kawari Road | *An. culicifacies* |  |
|  |  |  |  | Punjab; Lahore, Cantt |  |  |
|  |  |  |  | Quetta; Pashtun Abad, Balochistan, Marriabad, Sirki |  |  |
|  |  |  |  | Quetta; Pashtun Abad, Satellite Town | *An. stephensi* |  |
| Mukhtar, M.  (209) | 2006 | 2001-2 | Pakistan | Punjab; Faisalabad | *An. subpictus* |  |
| Ali, N.  (210) | 2007 | 1999-2000 | Pakistan | Punjab; Bahawalnagar, Haroonabad, Azhakhel Afghan Refugee Camp, | *An. stephensi* |  |
| Ali, N.  (211) | 2013 | 2010 | Pakistan | Khyber Pakhtunkhwa; Malakand, , Swat Ranizai | *An. stephensi, An. fluviatilis, An. maculatus, An. culicifacies, An. subpictus, An. lindesayi* |  |
| Khan, S. Y.  (212) | 2013 |  | Pakistan | Punjab; Gujrat, Jaman | *An. stephensi* |  |
|  |  |  |  | Punjab; Lahore, Mohlanwal |  |  |
|  |  |  |  | Punjab; Khushab, Noorpur Thal |  |  |
| Naz, S.  (213) | 2013 |  | Pakistan | Punjab; Okara | *An. culicifacies, An. stephensi* |  |
| Rathor, H. R.  (214) | 2013 | 2011 | Pakistan | Punjab; Layyah, Muzaffarpur, Dera Ghazi Khan, Rajanpur District | *An. stephensi* |  |
|  |  |  |  | Punjab; Layyah, Dera Ghazi Khan, Rajanpur District | *An. culicifacies* |  |
| Qasim, M.  (215) | 2014 | 212-13 | Pakistan | Punjab; Murree Hills | *An. stephensi, An. maculatus, An. theobaldi, An. fluviatilis* |  |
| Rana, S. M.  (216) | 2014 |  | Pakistan | Punjab; Muzaffargarh | *An. stephensi, An. culicifacies, An. fluviatilis, An. superpictus, An. subpictus* |  |
| Shaikh, S.  (217) | 2014 | 2009 | Pakistan | Karachi, Bin Qasim, Sindh, Faisal Cantonment, Karachi, Gadap, Gulshan-e-Iqbal, Jamshed Town, Korangi, Korangi town residency, Malir Cantonment, North Karachi Twp, Shah Faisal Town, Sindh Industrial Trading Estate | *An. culicifacies* |  |
|  |  |  |  | Sindh, Karachi, Gadap, Gulshan-e-Iqbal | *An. superpictus* |  |
|  |  |  |  | Sindh, Karachi, Gulshan-e-Iqbal, Korangi town residency, Malir Cantonment, North Karachi Twp, Orangi Town, Sindh Industrial Trading Estate | *An. stephensi* |  |
|  |  |  |  | Sindh, Karachi, North Karachi Twp, Orangi Town | *An. subpictus* |  |
| Ali, N.  (218) | 2015 | 2007 | Pakistan | Khyber Pakhtunkhwa; Charsadda, Dhakki, Totaki, Mathra | *An. splendidus, An. stephensi, An. stephensi* |  |
| Bibi, M.  (219) | 2015 |  | Pakistan | Punjab; Sialkot | *An. subpictus* |  |
| Oneeb, M.  (220) | 2015 |  | Pakistan | Punjab; Kasur, Shiekupura | *An. stephensi* |  |
| Manzoor, F.  (221) | 2020 | 2014-15 | Pakistan | Punjab; Lahore | *An. annularis, An. pulcherrimus, An. subpictus, An. nigerrimus, An. culicifacies, An. stephensi* |  |
| Attaullah, M.  (222) | 2021 | 2018-19 | Pakistan | Khyber Pakhtunkhwa; Malakand | *An. stephensi, An. maculatus, An. fluviatilis, An. subpictus, An. dthali, An. culicifacies, An. pallidus, An. annularis* |  |
|  |  |  |  | Khyber Pakhtunkhwa; Dir Lower | *An. stephensi, An. maculatus, An. fluviatilis, An. subpictus, An. dthali, An. culicifacies, An. pallidus, An. annularis* |  |
| Mehmood, A.  (223) | 2022 | 2014-16 | Pakistan | Punjab; Jhelum | *An. maculatus, An. tesselatus, An. theobaldi, An. stephensi, An. annularis, An. culicifacies* |  |
| Kardousha, M. M.  (224) | 2015 | 2009-11 | Qatar | Al Khor | *An. stephensi* |  |
| Peffly, R. L.  (225) | 1959 | 1954 | Saudi Arabia | Eastern; al-Ajam, safwa, Qatif, al-Hasa, Ain As Saih | *An. stephensi* |  |
|  |  |  | Saudi Arabia | Eastern; Qatif, Al Awjam, safwa | *An. pulcherrimus* |  |
|  |  |  | Saudi Arabia | Eastern; al-Mutairifi, Safwa | *An. coustani* |  |
|  |  |  | Saudi Arabia | Eastern; Ain al-Saih, Dammam | *An. fluviatilis* |  |
| Abdoon, A. M. M. O.  (226) | 2003 | 2000 | Saudi Arabia | Aseer Province; Al Birk | *An. rupicolus, An. dthali, An. sergenti, An. turkhudi* |  |
|  |  |  |  | Aseer Province; Wadi Ibn Hashbal | *An. turkhudi, An. multicolor* |  |
|  |  |  |  | Aseer Province; Al Qahma | *An. dthali, An. sergenti* |  |
|  |  |  |  | Aseer Province; Al Farshah | *An. pretoriensis, An. turkhudi, An. multicolor, An. rupicolus, An. dthali, An. sergenti, An. arabiensis* |  |
|  |  |  |  | Aseer Province; Al Majardah, Muhayil | *An. turkhudi, An. rupicolus, An. dthali, An. sergenti, An. arabiensis* |  |
|  |  |  |  | the Rijal Almaa province; Asir Region, Rijal Almaa | *An. turkhudi, An. multicolor, An. rupicolus, An. dthali, An. arabiensis* |  |
|  |  |  |  | Aseer Province; Marabah | *An. turkhudi, An. multicolor, An. dthali, An. arabiensis* |  |
| Al Ahmed, A. M.  (227) | 2010 | 2004-6 | Saudi Arabia | Al Bahah | *An. cinereus, An. dthali, An. stephensi, An. subpictus, An. turkhudi* |  |
| Ahmed, A. M.  (228) | 2011 | 2009-10 | Saudi Arabia | Al Ahsa | *An. multicolor* |  |
|  |  |  |  | Dammam | *An. tenebrosus* |  |
|  |  |  |  | Abha, Madinah, Najran, Taif | *An. cinereus* |  |
|  |  |  |  | Abha, Makkah, Najran | *An. culicifacies* |  |
|  |  |  |  | Abha, Al Bahah, Jeddah, Makkah, Najran, Taif | *An. dthali* |  |
|  |  |  |  | Abha, Al Bahah, Jeddah, Makkah, Najran | *An. gambiae* |  |
|  |  |  |  | Abha, Dammam, Al Ahsa, Jeddah, Makkah, Najran | *An. multicolor* |  |
|  |  |  |  | Abha, Dammam, Al Ahsa, Madinah, Makkah, Najran | *An. pretoriensis* |  |
|  |  |  |  | Abha, Dammam, Al Bahah, Jeddah, Makkah, Najran, Taif | *An. sergenti* |  |
|  |  |  |  | Abha, Dammam, Al Ahsa, Madinah, Jeddah, Makkah, Najran, Taif, Wadi ad-Dawasir | *An. stephensi* |  |
|  |  |  |  | Abha, Dammam, Al Ahsa, Al Bahah, Jeddah, Makkah, Najran, Taif | *An. subpictus* |  |
|  |  |  |  | Abha, Najran | *An. turkhudi* |  |
| Al-Sheik, A. A.  (229) | 2011 | 2007 | Saudi Arabia | Tihamah | *An. gambiae, An. dthali, An. pretoriensis, An. multicolor, An. rhodesiensis, An. turkhudi* |  |
| Alahmed, A. M.  (230) | 2012 | 2004-6 | Saudi Arabia | Al Ahsa | *An. cinereus, An. dthali, An. fluviatilis, An. gambiae, An. multicolor, An. pretoriensis* |  |
|  |  |  |  | Dammam | *An. cinereus, An. dthali, An. multicolor, An. pretoriensis* |  |
| Khater, E. I.  (231) | 2013 | 2009-10 | Saudi Arabia | Makkah, Jeddah, Al Bahah, Jezan | *An. dthali* |  |
|  |  |  |  | Makkah, Jeddah, Makkah, Arafat - Ground, Al Bahah, Jezan | *An. arabiensis* |  |
|  |  |  |  | Makkah, Jeddah, Al Bahah, | *An. sergenti* |  |
|  |  |  |  | Jezan | *An. azaniae, An. superpictus, An. multicolor* |  |
|  |  |  |  | Al Bahah, Jezan | *An. turkhudi* |  |
| Al Ashry, H. A.  (232) | 2014 |  | Saudi Arabia | Asir province | *An. dthali* |  |
| Mahyoub, J. A.  (233) | 2015 | 2010-11 | Saudi Arabia | Makkah | *An. dthali* |  |
| Alsheikh, A. A.  (234) | 2017 |  | Saudi Arabia | Jazan Region, Al-Khobah, Harub | *An. arabiensis* |  |
| Hassan, M. I.  (235) | 2017 | 2013-14 | Saudi Arabia | Taif, Makkah, Jeddah, | *An. dthali* |  |
|  |  |  |  | Taif | *An. sergenti* |  |
|  |  |  |  | Tabuk | *An. fluviatilis* |  |
| Shaalan, E. A. S.  (236) | 2017 | 2010-2011 | Saudi Arabia | Al Ahsa | *An. multicolor* |  |
| Munawar, K.  (237) | 2020 | 2013-16 | Saudi Arabia | Al Ahsa | *An. stephensi, An. rhodesiensis, An. cinereus* |  |
|  |  |  |  | Dammam | *An. stephensi* |  |
|  |  |  |  | Jeddah | *An. dthali* |  |
|  |  |  |  | Jezan | *An. arabiensis, An. dthali, An. cinereus* |  |
| Alghamdi, T. S.  (238) | 2021 | 2018 | Saudi Arabia | Taif | *An. dthali, An. arabiensis* |  |
| Ali, S.  (239) | 2022 | 2020 | Somalia | Berbera, Lawyacado, Borama, Sheikh Ali Jawhar, Daarbuduq, Hargeisa, Animal park, Daami | *An. stephensi* |  |
| Lewis, D. J.  (240) | 1937 | 1937 | Sudan | Jebel Moya | *An. myzomyia* |  |
| Omer, S. M.  (241) | 1968 | 1968 | Sudan | Omdurman | *An. gambiae* |  |
| Haridi, A. M.  (242) | 1972 | 1967 | Sudan | Khashm el Girba | *An. gambiae* |  |
| Akiyama, J.  (243) | 1973 | 1970 | Sudan | Kassala, Khashm el Girba | *An. gambiae* |  |
| Hemingway, J.  (244) | 1983 | 1978 | Sudan | Gezira | *An. arabiensis* |  |
| Mahmoud, A. A.  (245) | 1985 |  | Sudan | Al Jazirah | *An. arabiensis* |  |
| Dukeen, M. Y. H.  (246) | 1986 | 1978-79 | Sudan | Wadi Halfa, Wadi Halfa | *An. arabiensis* |  |
| el Safi, S. H.  (19) | 1986 |  | Sudan | Al Jazirah | *An. arabiensis* |  |
| El Sayed, B. B.  (247) | 2000 | 1995-96 | Sudan | Khartoum, Al Manshiya | *An. arabiensis* |  |
| Petrarca, V.  (248) | 2000 | 1974-87 | Sudan | Northern Sudan, Ungor Island2, PortSudan, Zeidab, Khartoum, Soba-Gereif West, Khashm-el-Girba, Khashm-el-Girba-1, New Halfa, Andala-1, HillatHakoma, Kassala, Showak, Gezira Irrigated, Ibrahim, Gezira area, Sennar Area, Alikifak, Abdin Gibir-1, Barakat, Wad Eleigili, Kosti area, Kosti town, Western Sudan, Er Rahad, Southern Sudan, Juba Town, Wau | *An. arabiensis* |  |
|  |  |  |  | Sennar Area, Sennar, Southern Sudan, Juba Town, Wau | *An. gambiae* |  |
| Hamad, A. A.  (249) | 2002 | 1994-95 | Sudan | Asar, Daraweesh | *An. arabiensis, An. pharoensis, An. funestus* |  |
| Himeidan, Y. E.  (250) | 2004 | 1999-2000 | Sudan | New Halfa | *An. arabiensis* |  |
| Himeidan, Y. E.  (251) | 2007 | 2005 | Sudan | New Halfa, Khashm el Girba, Kassala | *An. arabiensis* |  |
| Matambo, T. S.  (252) | 2007 | 2005 | Sudan | Sennar | *An. arabiensis* |  |
| Abdalla, H.  (253) | 2008 | 2005-6 | Sudan | Gezira, Rufaa, Wad Rawa | *An. arabiensis* |  |
| Bashir, A.  (254) | 2008 | 2005 | Sudan | Khartoum | *An. arabiensis* |  |
| Helinski, M. E.  (255) | 2008 | 2004 | Sudan | Khartoum | *An. arabiensis* |  |
| Himeidan, Y. E.  (256) | 2008 |  | Sudan | New Halfa | *An. arabiensis* |  |
| Ageep, T. B.  (257) | 2009 | 2005-7 | Sudan | Merowe Dam | *An. arabiensis* |  |
| Elimam, A. M.  (258) | 2009 | 2005-7 | Sudan | Khartoum North, Shambat | *An. arabiensis* |  |
| Ranson, H.  (259) | 2009 | 2008-9 | Sudan | Wad Medani, Al Manaqil, Wad el Haddad, Rufaa | *An. gambiae* |  |
| Himeidan, Y. E.  (260) | 2011 | 2005-6 | Sudan | Koka, Salala | *An. arabiensis, An. rufipes* |  |
| Himeidan, Y. E.  (261) | 2011 | 2009 | Sudan | Sennar, Omdurman, Al Bostan, Mayirno, Ad-Damazīn, Ar Rusayris, Kosti, Rabak, AsalayaKhartoum, Al-Remila, Khartoum, Al-Kalakla, Al Qadarif, Al Faw, Showak | *An. arabiensis* |  |
| Seidahmed, O. M.  (262) | 2012 | 2007 | Sudan | Khartoum | *An. arabiensis* |  |
| Abuelmaali, S. A.  (263) | 2013 | 2010-11 | Sudan | khartoum, SOBA West, Al-Remila, Tuti Island, Althamaniat, Algamoyia, Alseliat | *An. arabiensis* |  |
| Aboud, M.  (264) | 2014 | 1998-99-2001-2004-2006 | Sudan | Khartoum, Al Qadarif, Kassala | *An. arabiensis* |  |
| Hamza, A. M.  (265) | 2016 | 2000-2 | Sudan | Kassala | *An. arabiensis* |  |
| Azrag, R. S.  (266) | 2018 | 2015 | Sudan | Khartoum | *An. arabiensis* |  |
| Ismail, B. A.  (267) | 2018 | 2010-14 | Sudan | El Hoosh, Hag 'Abdullah, Gallabat, New Halfa, | *An. arabiensis* |  |
|  |  |  |  | Hag 'Abdullah | *An. gambiae* |  |
| Korti, M. Y.  (268) | 2021 |  | Sudan | Al-hamadab, Merowe | *An. arabiensis* |  |
| Hamza, A. M.  (269) | 2023 | 2022 | Sudan | Kassala | *An. arabiensis* |  |
| Cropper, J.  (170) | 1902 | 1900 | Syria | Baniyas | *An. superpictus, An. maculipennis* |  |
| Onori, E.  (270) | 1972 | 1968 | Syria | Tangier, Ghab area | *An. sacharovi* |  |
| Krida, G.  (271) | 1998 |  | Tunisia | Rades, Mencher | *An. labranchiae* |  |
|  |  |  |  | Meknassy | *An. sergenti* |  |
|  |  |  |  | Sidi Bouzid | *An. multicolor* |  |
| Tabbabi, A.  (272) | 2015 | 2012 | Tunisia | Bizerte Governorate; Biezrte, Ariana, Ben Arous, Beja, Jandouba, Kairouan, Monastir | *An. labranchiae* |  |
|  |  |  |  | Tozeur | *An. algeriensis, An. multicolor* |  |
|  |  |  |  | Tataouine | *An. sergenti, An. multicolor* |  |
| Maffi, M.  (273) | 1971 | 1971 | Yemen | Ibb Governorate;Al Wadi, Al Udayn District | *An. minimus, An. funestus* |  |
| Kravchenko, V. K.  (274) | 1979 |  | Yemen | Hadhramaut Governorate, | *An. sergenti, An. dthali* |  |
| Al-Maktari, M. T.  (275) | 1999 |  | Yemen | Zabid | *An. arabiensis, An. culicifacies, An. rhodesiensis* |  |
| Allan, R.  (276) | 2023 | 2021 | Yemen | Aden | *An. stephensi* |  |

REFERENCES:

1. Büttiker W. Observations on the physiology of adult anophelines in Asia. Bull World Health Organ. 1958;19(6):1063-71.

2. Dukhanina NN, Nushin MK, Polevoy NI. Malaria problem and malaria control measures in north North I. Malaria in North Afganistan (Russian). Meditsinskaya Parazitologiya i Parazitarnye Bolezni. 1975;44(3):333-7.

3. Onori E, Nushin MK, Cullen JE, Yakubi GH, Mohammed K, Christal FA. An epidemiological assessment of the residual effect of DDT on Anopheles hyrcanus sensulato and A. Pulcherrimus (Theobold) in the North eastern Region of Afghanistan. Transactions of the Royal Society of Tropical Medicine and Hygiene. 1975;69(2):236-42.

4. Faulde MK, Scharninghausen JJ, Tisch M. Fire fighting truck-based emergency mosquito biolarviciding to prevent outbreaks of malaria and arboviral disease in Kabul, Afghanistan. Journal of Pest Science. 2008;81(2):71-7.

5. Hoosh-Deghati H, Dinparast-Djadid N, Moin-Vaziri V, Atta H, Raz AA, Seyyed-Tabaei SJ, et al. Composition of Anopheles species collected from selected malarious areas of Afghanistan and Iran. Journal of Arthropod-Borne Diseases. 2017;11(3):354-62.

6. Safi NHZ, Ahmadi AA, Nahzat S, Warusavithana S, Safi N, Valadan R, et al. Status of insecticide resistance and its biochemical and molecular mechanisms in Anopheles stephensi (Diptera: Culicidae) from Afghanistan. Malaria Journal. 2019;18(1).

7. Rodhain F. [Preliminary results of an entomological survey of the potential arbovirus vectors in the French Territory of Afars and Issas]. Bull Soc Pathol Exot Filiales. 1976;69(2):169-74.

8. Carteron B, Morvan D, Rodhain F. The question of endemic malaria in Republic of Djibouti. Medecine Tropicale. 1978;38(3):299-304.

9. Faulde MK, Rueda LM, Khaireh BA. First record of the Asian malaria vector Anopheles stephensi and its possible role in the resurgence of malaria in Djibouti, Horn of Africa. Acta Tropica. 2014;139:39-43.

10. Shousha AT. Species-eradication: The Eradication of Anopheles gambioe from Upper Egypt, 1942-1945. Bull World Health Organ. 1948;1(2):309-52.

11. Abdel-Malek AA. STUDY OF THE FEEDING HABITS OF MALE ANOPHELES SERGENTI THEO. AT SIWA OASIS USING RADIOPHOSPHORUS. Bulletin of the World Health Organization. 1964;30:137-9.

12. abdel-Malek AA, abdel-Aal MA. Study of the dispersion and flight range of Anopheles sergenti Theo. in Siwa Oasis using radioactive isotopes as markers. Bulletin of the World Health Organization. 1966;35(6):968-73.

13. Kamel OM, Gad AM. Present status of susceptibility of common UAR anopheline species to insecticides. The Journal of the Egyptian Public Health Association. 1966;41(5):263-74.

14. Soliman AA, Rifaat MA, Ibrahim MT. Biology of Egyptian anophelines. 1. Biological activities of Anopheles pharoensis in nature. Journal of Tropical Medicine and Hygiene. 1967;70(3):63-7.

15. Abdel-Malek AA, Tantawy AO, Wakid AM. Studies on the eradication of Anopheles pharoensis by the sterile-male technique using cobalt-60. VII. Population dynamics of mosquitoes in Fayoum area, UAR. Journal of economic entomology. 1969;62(2):348-51.

16. Shalaby AM. Changes in the Ovaries of Anopheles multicolor and Anopheles pharoensis (Diptera: Culicidae) Following Oviposition. Zeitschrift für Angewandte Entomologie. 1971;69(1-4):187-97.

17. El‐Gayar FH, Gawaad AAA, Watson WM. Development of a Standardized Technique for Rearing Larvae of Anopheles pharoensis Theo. (Dipt., Culicidae), and Preparation of Dosage‐Mortality Curves for five Larvicides. Zeitschrift für Angewandte Entomologie. 1972;70(1-4):316-22.

18. Gad AM, el Said S, Hassan AN. Ecology of Anopheles (Cellia) Sergent II Theobald in the eastern desert, Red Sea Governorate, Egypt. Journal of the Egyptian Society of Parasitology. 1984;14(1):1-6.

19. el Safi SH, Haridi AM. Field trial of the insect growth regulator, Dimilin, for control of Anopheles pharoensis in Gezira, Sudan. Journal of the American Mosquito Control Association. 1986;2(3):374-5.

20. Kenawy M, Zimmerman JH, Beier JC, el Said S, Abbassy MM. Host-feeding patterns of Anopheles sergentii and An. multicolor (Diptera: Culicidae) in Siwa and El Gara oases, Egypt. Journal of medical entomology. 1986;23(5):576-7.

21. Beier JC, Zimmerman JH, Kenawy MA, Elsaid S, Abbassy MM. HOST-FEEDING PATTERNS OF THE MOSQUITO COMMUNITY (DIPTERA, CULICIDAE) IN 2 FAIYUM GOVERNORATE VILLAGES, EGYPT. Journal of Medical Entomology. 1987;24(1):28-34.

22. Gad AM, el Said S, Hassan AN, Shoukry A. The distribution and ecology of the mosquitoes in the Red Sea Governorate, Egypt. Journal of the Egyptian Society of Parasitology. 1987;17(1):207-21.

23. Hilmy NM, Merdan AI, Ibrahim AA. Mosquito distribution in Qaluobiya Governorate, Egypt. Journal of the Egyptian Society of Parasitology. 1987;17(1):223-31.

24. Omar AH, Shaurub EH. Mating behaviour and insemination in Anopheles multicolor Cambouliu. J Egypt Soc Parasitol. 1987;17(1):381-7.

25. Zimmerman JH, Abbassy MM, Hanafi HA, Beier JC, Dees WH. HOST-FEEDING PATTERNS OF MOSQUITOS (DIPTERA, CULICIDAE) IN A RURAL VILLAGE NEAR CAIRO, EGYPT. Journal of Medical Entomology. 1988;25(5):410-2.

26. Kenawy MA. Development and survival of Anopheles pharoensis and An. multicolor from Faiyum, Egypt. Journal of the American Mosquito Control Association. 1991;7(4):551-5.

27. Kenawy MA, Mohamed M, Hamed MS, Merdan AI. Comparison of the life table characteristics of Anopheles sergentii (Diptera: Culicidae) from two malarious areas in Egypt. The Journal of the Egyptian Public Health Association. 1995;70(3-4):323-41.

28. Beavers GM, Hanafi HA, Tetreault GE. Response of mosquitoes (Diptera: Culicidae) to carbon dioxide and octenol in Egypt. Journal of the Egyptian Society of Parasitology. 1998;28(2):303-12.

29. Bassiouny HK, Awad OM, Ahmed MH. Bionomics of the anopheline vectors in an endemic area in Fayoum Governorate, Egypt. The Journal of the Egyptian Public Health Association. 1999;74(3-4):241-61.

30. Kenawy MA, Sowilem MM, Abdel-Hamid YM, Wahba MM. Preliminary observations on cross-mating of the malaria vector, Anopheles sergentii from two Egyptian oases. Journal of the Egyptian Society of Parasitology. 2000;30(3):761-4.

31. Hassan AN, Onsi HM. Remote sensing as a tool for mapping mosquito breeding habitats and associated health risk to assist control efforts and development plans: a case study in Wadi El Natroun, Egypt. Journal of the Egyptian Society of Parasitology. 2004;34(2):367-82.

32. Abdel-Hamid YM, Soliman MI, Kenawy MA. Mosquitoes (Diptera: Culicidae) in relation to the risk of disease transmission in El Ismailia Governorate, Egypt. Journal of the Egyptian Society of Parasitology. 2011;41(2):347-56.

33. El-Bahnasawy MM, Saleh NM, Khalil MF, Morsy TA. The impact of three anopheline mosquito species in Toshka, on the introduction of chloroquine resistant P. falciparum to Egypt. Journal of the Egyptian Society of Parasitology. 2011;41(3):573-92.

34. Ammar SE, Kenawy MA, Abdel-Rahman HA, Gad AM, Hamed AF. Ecology of the mosquito larvae in urban environments of Cairo Governorate, Egypt. Journal of the Egyptian Society of Parasitology. 2012;42(1):191-202.

35. Bahgat IM. Impact of physical and chemical characteristics of breeding sites on mosquito larval abundance at Ismailia Governorate, Egypt. Journal of the Egyptian Society of Parasitology. 2013;43(2):399-406.

36. Dahesh SM, Mostafa HI. REEVALUATION OF MALARIA PARASITES IN EL-FAYOUM GOVERNORATE, EGYPT USING RAPID DIAGNOSTIC TESTS (RDTS). Journal of the Egyptian Society of Parasitology. 2015;45(3):617-28.

37. Mahmoud DM, Hussein HM, El Gozamy BMR, Thabet HS, Hassan MA, Meselhey RA. Screening of Plasmodium parasite in vectors and humans in three villages in Aswan Governorate, Egypt. Journal of parasitic diseases : official organ of the Indian Society for Parasitology. 2019;43(1):158-63.

38. El Ela AAA, Mostafa A, Ahmed EA, Khalil A, Ghonaim M, Ahmed AM. Mosquito abundance and physicochemical characteristics of their breeding water in El-Fayoum Governorate, Egypt. Journal of King Saud University Science. 2024;36(2).

39. Mohamed HA, Gad HA, Oraby HK. Mosquito species composition and their relative abundance in Beni-Suef Governorate, Egypt. International Journal of Tropical Insect Science. 2024.

40. Garrett-Jones C. An experiment in trapping and controlling Anopheles maculipennis in North Iran. Bulletin of the World Health Organization. 1951;4(4):547-62.

41. De Zulueta J, Jolivet P, Thymakis K, Caprari P. Seasonal variations in susceptibility to DDT of Anopheles maculipennis in Iran. Bulletin of the World Health Organization. 1957;16(2):475-9.

42. De Zulueta J. Insecticide resistance in Anopheles sacharovi. Bull World Health Organ. 1959;20(5):797-822.

43. Golestani J. Evaluation of bromophos‐ethyl for the control of Anopheles Sp. In Southern iran. Pesticide Science. 1970;1(3):81-3.

44. Motabar M, Mofidi C, Saebi A, Ghiassedin M, Rouhani F. A medicated salt project in the Kazeroun area, Iran--1962-1967. Journal of Tropical Medicine and Hygiene. 1971;74(2):39-44.

45. Manoochehri A, Ghiasseddin M, Shahgudian ER. Anopheles dthali patton, i 905, a new secondary vector in southern iran. Annals of Tropical Medicine and Parasitology. 1972;66(4):537-8.

46. Eshghy N, Mesghali A, Behbahani G, Motabar M. Area scale evaluation of Sumithion (OMS 43) for control of adult anopheline mosquitoes in Mamasani, Kazeroun, Southern Iran, 1972. Iranian Journal of Public Health. 1973;2(1):14-21.

47. Zaini A, Manouchehri AV. Preliminary notes on the development of DDT resistance in Anopheles culicifacies Giles in Baluchestan Province, Southern Iran. Iranian Journal of Public Health. 1973;2(3):156-62.

48. Javadian E, Acheson MA. An evaluation of the effect of larviciding operations in rural areas near Abadan, Iran. Iranian Journal of Public Health. 1974;3(1):54-61.

49. Manouchehri AV, Zaini A, Javadian E, Saebi E. Resistance of Anopheles sacharovi Favre to DDT in Iran, 1973. Iranian Journal of Public Health. 1974;2(4):204-11.

50. Sahabi Z, Amirkhanian JD, Shahgoudian E. The polytene chromosomal pattern of Anopheles stephensi mysorensis of Kazeroon (Iran). Iranian Journal of Public Health. 1974;2(4):194-8.

51. Manouchehri AV, Djanbakhsh B, Eshghi N. The biting cycle of Anopheles dthali. A. fluviatilis and A. stephensi in southern Iran. Tropical and Geographical Medicine. 1976;28(3):224-7.

52. Zaim M, Ershadi MR, Manouchehri AV, Hamdi MR. The use of CDC light traps and other procedures for sampling malaria vectors in southern Iran. Journal of the American Mosquito Control Association. 1986;2(4):511-5.

53. Manouchehri AV, Yaghoobi-Ershadi MR. Propoxur susceptibility test of Anopheles stephensi in southern Islamic Republic of Iran (1976-86). Journal of the American Mosquito Control Association. 1988;4(2):159-62.

54. Weiser J, Zaim M, Saebi E. Coelomomyces irani sp.n. infecting Anopheles maculipennis in Iran. Journal of Invertebrate Pathology. 1991;57(2):290-1.

55. Zaim M, Javaherian Z. Occurrence of Anopheles culicifacies species A in Iran. Journal of the American Mosquito Control Association. 1991;7(2):324-6.

56. Zaim M, Manouchehri AV, Motabar M, Mowlaii G, Kayedi MH, Pakdad P, et al. Ecology of Anopheles pulcherrimus in Baluchistan, Iran. Journal of the American Mosquito Control Association. 1992;8(3):293-6.

57. Zaim M, Subbarao SK, Manouchehri AV, Cochrane AH. Role of Anopheles culicifacies s.l. and An. pulcherrimus in malaria transmission in Ghassreghand (Baluchistan), Iran. Journal of the American Mosquito Control Association. 1993;9(1):23-6.

58. Zaim M, Manouchehri AV, Motabar M, Emadi AM, Nazari M, Pakdad K, et al. Anopheles culicifacies in Baluchistan, Iran. Medical and Veterinary Entomology. 1995;9(2):181-6.

59. Yaghoobi-Ershadi MR, Namazi J, Piazak N. Bionomics of Anopheles sacharovi in Ardebil province, northwestern Iran during a larval control program. Acta Tropica. 2001;78(3):207-15.

60. Naddaf SR, Oshaghi MA, Vatandoost H, Assmar M. Molecular characterization of Anopheles fluviatilis species complex in the Islamic Republic of Iran. Eastern Mediterranean Health Journal. 2003;9(3):257-65.

61. Sedaghat MM, Linton YM, Oshaghi MA, Vatandoost H, Harbach RE. The Anopheles maculipennis complex (Diptera: Culicidae) in Iran: Molecular characterization and recognition of a new species. Bulletin of Entomological Research. 2003;93(6):527-35.

62. Vatandoost H, Shahi H, Abai MR, Hanafi-Bojd AA, Oshaghi MA, Zamani G. Larval habitats of main malaria vectors in Hormozgan province and their susceptibility to different larvicides. The Southeast Asian journal of tropical medicine and public health. 2004;35 Suppl 2:22-5.

63. Basseri HR, Moosakazemi SH, Yosafi S, Mohebali M, Hajaran H, Jedari M. Anthropophily of malaria vectors in Kahnouj district, south of Kerman, Iran. Iranian Journal of Public Health. 2005;34(2):27-35.

64. Ghavami MB. Estimation and comparison of Anopheles maculipennis s.l. (Diptera: Culicidae) survival rates with light-trap and indoor resting data. Iranian Journal of Public Health. 2005;34(2):48-57.

65. Vatandoost H, Mashayekhi M, Abaie MR, Aflatoonian MR, Hanafi-Bojd AA, Sharifi I. Monitoring of insecticides resistance in main malaria vectors in a malarious area of Kahnooj district, Kerman province, southeastern Iran. Journal of Vector Borne Diseases. 2005;42(3):100-8.

66. Davari B, Vatandoost H, Ladonni H, Shaeghi M, Oshaghi MA, Basseri HR, et al. Comparative efficacy of different imagicides against different strains of Anopheles stephensi in the malarious areas of Iran, 2004-2005. Pakistan Journal of Biological Sciences. 2006;9(5):885-92.

67. Djadid ND, Gholizadeh S, Aghajari M, Zehi AH, Raeisi A, Zakeri S. Genetic analysis of rDNA-ITS2 and RAPD loci in field populations of the malaria vector, Anopheles stephensi (Diptera: Culicidae): Implications for the control program in Iran. Acta Tropica. 2006;97(1):65-74.

68. Khoobdel M, Shayeghi M, Vatandoost H, Rassi Y, Abaei MR, Ladonni H, et al. Field evaluation of permethrin-treated military uniforms against Anopheles stephensi and 4 species of Culex (Diptera:Culicidae) in Iran. Journal of Entomology. 2006;3(2):108-18.

69. Vatandoost H, Oshaghi MA, Abaie MR, Shahi M, Yaaghoobi F, Baghaii M, et al. Bionomics of Anopheles stephensi Liston in the malarious area of Hormozgan province, southern Iran, 2002. Acta Tropica. 2006;97(2):196-203.

70. Abai MR, Azari-Hamidian S, Ladonni H, Hakimi M, Mashhadi-Esmail K, Sheikhzadeh K, et al. Fauna and Checklist of Mosquitoes (Diptera: Culicidae) of East Azerbaijan Province, Northwestern Iran. Iranian Journal of Arthropod-Borne Diseases. 2007;1(2):27-33.

71. Djadid ND, Forouzesh F, Karimi M, Raeisi A, Hassan-Zehi A, Zakeri S. Monitoring pyrethroid insecticide resistance in major malaria vector Anopheles culicifacies: Comparison of molecular tools and conventional susceptibility test. Iranian Biomedical Journal. 2007;11(3):169-76.

72. Djadid ND, Gholizadeh S, Tafsiri E, Romi R, Gordeev M, Zakeri S. Molecular identification of Palearctic members of Anopheles maculipennis in northern Iran. Malaria Journal. 2007;6.

73. Moosa-Kazemi SH, Vatandoost H, Raeisi A, Akbarzadeh K. Deltamethrin Impregnated Bed Nets in a Malaria Control Program in Chabahar, Southeast Baluchistan, IR Iran. Iranian Journal of Arthropod-Borne Diseases. 2007;1(1):43-51.

74. Oshaghi MA, Shemshad K, Yaghobi-Ershadi MR, Pedram M, Vatandoost H, Abaie MR, et al. Genetic structure of the malaria vector Anopheles superpictus in Iran using mitochondrial cytochrome oxidase (COI and COII) and morphologic markers: A new species complex? Acta Tropica. 2007;101(3):241-8.

75. Vatandoost H, Shahi M, Hanafi-Bojd AA, Abai MR, Oshaghi MA, Raffi F. Ecology of <i>Anopheles dthali</i> Patton in Bandar Abbas District, Hormozgan Province, Southern Iran. Iranian Journal of Arthropod-Borne Diseases. 2007;1(1):21-7.

76. Abai MR, Mehravaran A, Vatandoost H, Oshaghi MA, Javadian E, Mashayekhi M, et al. Comparative performance of imagicides on Anopheles stephensi, main malaria vector in a malarious area, southern Iran. Journal of Vector Borne Diseases. 2008;45(4):307-12.

77. Basseri HR, Doosti S, Akbarzadeh K, Nateghpour M, Whitten MM, Ladoni H. Competency of Anopheles stephensi mysorensis strain for Plasmodium vivax and the role of inhibitory carbohydrates to block its sporogonic cycle. Malar J. 2008;7:131.

78. Dinparast Djadid N, Barjesteh H, Forouzesh F, Zakeri S. Determination of glutathione S-transferase e2 region (GSTe2) in DDT susceptible and resistant Anopheles stephensi populations: Significance and application of nucleotide and amino acid comparison. Iranian Journal of Biotechnology. 2008;6(2):92-7.

79. Ghavami MB, Dinparast Djadid N, Haniloo A. Molecular characteristics of Anopheles maculipennis Meigen in Zanjan, North West of Iran, inferred from ITS2 sequence analysis. Pakistan Journal of Biological Sciences. 2008;11(4):539-45.

80. Rasoolian M, Sadrai J, Nikbakhtzadeh MR. Identification of the anopheles mosquitoes (diptera: Culicidae) of southern iran using analysis of cuticular hydrocarbons. Animal Cells and Systems. 2008;12(3):165-70.

81. Shemshad K, Oshaghi MA, Yaghoobi-Ershadi MR, Vatandoost H, Abaie MR, Akbarzadeh K, et al. Mitochondrial DNA (mtDNA) structure of Anopheles superpictus populations in Iran. Tehran University Medical Journal. 2008;65(1):24-32.

82. Djadid ND, Jazayeri H, Gholizadeh S, Rad Sh P, Zakeri S. First record of a new member of Anopheles Hyrcanus Group from Iran: molecular identification, diagnosis, phylogeny, status of kdr resistance and Plasmodium infection. J Med Entomol. 2009;46(5):1084-93.

83. Azari-Hamidian S, Linton YM, Abai MR, Ladonni H, Oshaghi MA, Hanafi-Bojd AA, et al. Mosquito (Diptera: Culicidae) fauna of the Iranian islands in the Persian Gulf. Journal of Natural History. 2010;44(15-16):913-25.

84. Basseri H, Raeisi A, Ranjbar Khakha M, Pakarai A, Abdolghafar H. Seasonal abundance and host-feeding patterns of anopheline vectors in malaria endemic area of Iran. Journal of Parasitology Research. 2010;2010.

85. Farzinnia B, Saghafipour A, Abai M. Malaria situation and anopheline mosquitoes in qom province, central iran. Iran J Arthropod Borne Dis. 2010;4(2):61-7.

86. Hasasan V, Hossein ZA. Responsiveness of Anopheles maculipennis to different imagicides during resurgent malaria. Asian Pacific Journal of Tropical Medicine. 2010;3(5):360-3.

87. Hassan V, Kamran A, Ali HBA, Minoo M, Mohammad S, Elfatih M, et al. Malaria stratification in a malarious area, a field exercise. Asian Pacific Journal of Tropical Medicine. 2010;3(10):807-11.

88. Nikookar S, Moosa-Kazemi S, Oshaghi M, Yaghoobi-Ershadi M, Vatandoost H, Kianinasab A. Species composition and diversity of mosquitoes in neka county, mazandaran province, northern iran. Iran J Arthropod Borne Dis. 2010;4(2):26-34.

89. Raeisi A, Abai M, Akbarzadeh K, Nateghpour M, Sartipi M, Hassanzehi A, et al. Residual Effects of Deltamethrin WG 25% as a New Formulation on Different Surfaces against Anopheles stephensi, in Southeastern Iran. Iran J Arthropod Borne Dis. 2010;4(1):60-5.

90. Shahandeh K, Basseri H, Pakari A, Riazi A. Mosquito vector biting and community protection in a malarious area, siahoo district, hormozgan, iran. Iran J Arthropod Borne Dis. 2010;4(2):35-41.

91. Ahmad M, Vatandoost H, Ali OM, Reza AM. Anopheline mosquitoes and their role for malaria transmission in an endemic area, southern Iran. Asian Pacific Journal of Tropical Disease. 2011;1(3):209-11.

92. Azari-Hamidian S. Larval habitat characteristics of the genus Anopheles (Diptera: Culicidae) and a checklist of mosquitoes in Guilan Province, northern Iran. Iranian Journal of Arthropod-Borne Diseases. 2011;5(1):37-53.

93. Azari-Hamidian S, Abai MR, Arzamani K, Bakhshi H, Karami H, Ladonni H, et al. Mosquitoes (diptera: Culicidae) of north khorasan province, northeastern iran and the zoogeographic affinities of the iranian and middle asian mosquito fauna. Journal of Entomology. 2011;8(3):204-17.

94. Hassan V, Arash R, Mehdi J, Ahmad R, Ali HB, Wali YA, et al. Demonstration of malaria situation analysis, stratification and planning in Minab District, southern Iran. Asian Pac J Trop Med. 2011;4(1):67-71.

95. Mehravaran A, Oshaghi MA, Vatandoost H, Abai MR, Ebrahimzadeh A, Roodi AM, et al. First report on Anopheles fluviatilis U in southeastern Iran. Acta Tropica. 2011;117(2):76-81.

96. Oshaghi MA, Vatandoost H, Gorouhi A, Abai MR, Madjidpour A, Arshi S, et al. Anopheline species composition in borderline of Iran-Azerbaijan. Acta Tropica. 2011;119(1):44-9.

97. Vatandoost H, Emami SN, Oshaghi MA, Abai MR, Raeisi A, Piazzak N, et al. Ecology of malaria vector Anopheles culicifacies in a malarious area of Sistan va Baluchestan province, south-east Islamic Republic of Iran. Eastern Mediterranean Health Journal. 2011;17(5):439-45.

98. Basseri HR, Abai MR, Raeisi A, Shahandeh K. Community sleeping pattern and anopheline biting in Southeastern Iran: A country earmarked for malaria elimination. American Journal of Tropical Medicine and Hygiene. 2012;87(3):499-503.

99. Hanafi-Bojd AA, Vatandoost H, Oshaghi MA, Charrahy Z, Haghdoost AA, Sedaghat MM, et al. Larval habitats and biodiversity of anopheline mosquitoes (Diptera: Culicidae) in a malarious area of southern Iran. Journal of Vector Borne Diseases. 2012;49(2):91-100.

100. Khoobdel M, Azari-Hamidian S, Hanafi-Bojd AA. Mosquito fauna (Diptera: Culicidae) of the Iranian islands in the Persian Gulf II. Greater Tonb, Lesser Tonb and Kish Islands. Journal of Natural History. 2012;46(31-32):1939-45.

101. Mehravaran A, Vatandoost H, Oshaghi MA, Abai MR, Edalat H, Javadian E, et al. Ecology of Anopheles stephensi in a malarious area, southeast of Iran. Acta Medica Iranica. 2012;50(1):61-5.

102. Saghafipour A, Abai M, Farzinnia B, Nafar R, Ladonni H, Azari-Hamidian S. Mosquito (Diptera: culicidae) fauna of qom province, iran. J Arthropod Borne Dis. 2012;6(1):54-61.

103. Vatandoost H, Abai MR. Irritability of malaria vector, Anopheles sacharovi to different insecticides in a malaria-prone area. Asian Pacific Journal of Tropical Medicine. 2012;5(2):113-6.

104. Vatandoost H, Hanafi-Bojd AA. Indication of pyrethroid resistance in the main malaria vector, Anopheles stephensi from Iran. Asian Pacific Journal of Tropical Medicine. 2012;5(9):722-6.

105. Banafshi O, Abai MR, Ladonni H, Bakhshi H, Karami H, Azari-Hamidian S. The fauna and ecology of mosquito larvae (Diptera: Culicidae) in western Iran. Turkish Journal of Zoology. 2013;37(3):298-307.

106. Nejati J, Vatandoost H, Oshghi MA, Salehi M, Mozafari E, Moosa-Kazemi SH. Some ecological attributes of malarial vector Anopheles superpictus Grassi in endemic foci in southeastern Iran. Asian Pacific Journal of Tropical Biomedicine. 2013;3(12):1003-8.

107. Shahi M, Hanafi-Bojd AA, Vatandoost H, Soleimani Ahmadi M. Susceptibility status of Anopheles stephensi liston the main malaria vector, to deltamethrin and bacillus thuringiensis in the endemic malarious area of Hormozgan province, southern Iran. Journal of Kerman University of Medical Sciences. 2013;20(1):87-95.

108. Soleimani-Ahmadi M, Vatandoost H, Hanafi-Bojd AA, Zare M, Safari R, Mojahedi A, et al. Environmental characteristics of anopheline mosquito larval habitats in a malaria endemic area in Iran. Asian Pacific Journal of Tropical Medicine. 2013;6(7):510-5.

109. Soltani A, Vatandoost H, Oshadhi MA, Enayati AA, Raeisi A, Eshraghian MR, et al. Baseline susceptibility of different geographical strains of Anopheles stephensi (diptera: Culicidae) to temephos in malarious areas of Iran. Journal of Arthropod-Borne Diseases. 2013;7(1):56-65.

110. Amani H, Yaghoobi-Ershadi MR, Kassiri H. The ecology and larval habitats characteristics of anopheline mosquitoes (Diptera: Culicidae) in Aligudarz County (Luristan province, western Iran). Asian Pacific Journal of Tropical Biomedicine. 2014;4:S233-S41.

111. Chavshin AR, Oshaghi MA, Vatandoost H, Pourmand MR, Raeisi A, Terenius O. Isolation and identification of culturable bacteria from wild Anopheles culicifacies, a first step in a paratransgenesis approach. Parasites and Vectors. 2014;7(1).

112. Khoshdel-Nezamiha F, Vatandoost H, Azari-Hamidian S, Bavani MM, Dabiri F, Entezar-Mahdi R, et al. Fauna and larval habitats of Mosquitoes (Diptera: Culicidae) of West Azerbaijan Province, Northwestern Iran. Journal of Arthropod-Borne Diseases. 2014;8(2):163-73.

113. Zahirnia AH, Zendehfili H. Mosquito fauna (Diptera: Culicidae) of Hamedan county, Western Iran. Journal of Arthropod-Borne Diseases. 2014;8(2):212-8.

114. Ataie A, Moosa-Kazemi SH, Vatandoost H, Yaghoobi-Ershadi MR, Bakhshi H, Anjomruz M. Assessing the susceptibility status of mosquitoes (diptera: Culicidae) in a dirofilariasis focus, Northwestern Iran. Journal of Arthropod-Borne Diseases. 2015;9(1):7-21.

115. Chavshin AR, Dabiri F, Vatandoost H, Bavani MM. Susceptibility of Anopheles maculipennis to different classes of insecticides in West Azarbaijan Province, Northwestern Iran. Asian Pacific Journal of Tropical Biomedicine. 2015;5(5):403-6.

116. Farhadinejad R, Mousavi M, Amraee K. The species composition of mosquitoes (Diptera: Culicidae) in the Mahshahr district, Khuzestan province, southwest of Iran. Archives of Razi Institute. 2015;70(2):89-95.

117. Fathian M, Vatandoost H, Moosa-Kazemi SH, Raeisi A, Yaghoobi-Ershadi MR, Oshaghi MA, et al. Susceptibility of Culicidae mosquitoes to some insecticides recommended by WHO in a malaria endemic area of Southeastern Iran. Journal of Arthropod-Borne Diseases. 2015;9(1):22-34.

118. Ladonni H, Azari-Hamidian S, Alizadeh M, Abai MR, Bakhshi H. The fauna, habitats, and affinity indices of mosquito larvae (Diptera: Culicidae) in Central Iran. North-Western Journal of Zoology. 2015;11(1):76-85.

119. Maghsoodi N, Ladonni H, Basseri HR. Species composition and seasonal activities of malaria vectors in an area at reintroduction prevention stage, Khuzestan, South-Western Iran. Journal of Arthropod-Borne Diseases. 2015;9(1):60-70.

120. Moosa-Kazemi SH, Zahirnia AH, Sharifi F, Davari B. The fauna and ecology of mosquitoes (Diptera: Culicidae) in Western Iran. Journal of Arthropod-Borne Diseases. 2015;9(1):49-59.

121. Nikookar SH, Moosa-Kazemi SH, Yaghoobi-Ershadi MR, Vatandoost H, Oshaghi MA, Ataei A, et al. Fauna and larval habitat characteristics of mosquitoes in Neka County, Northern Iran. Journal of Arthropod-Borne Diseases. 2015;9(2):253-66.

122. Soleimani-Ahmadi M, Vatandoost H, Zare M, Turki H, Alizadeh A. Topographical distribution of anopheline mosquitoes in an area under elimination programme in the south of Iran. Malaria Journal. 2015;14(1).

123. Abai MR, Hanafi-Bojd AA, Vatandoost H. Laboratory evaluation of temephos against Anopheles stephensi and Culex pipiens Larvae in Iran. Journal of Arthropod-Borne Diseases. 2016;10(4):510-8.

124. Gorouhi MA, Vatandoost H, Oshaghi MA, Raeisi A, Enayati AA, Mirhendi H, et al. Current susceptibility status of Anopheles stephensi (Diptera: Culicidae) to different imagicides in a Malarious Area, Southeastern of Iran. Journal of Arthropod-Borne Diseases. 2016;10(4):493-500.

125. Khoshdel-Nezamiha F, Vatandoost H, Oshaghi MA, Azari-Hamidian S, Mianroodi RA, Dabiri F, et al. Molecular characterization of mosquitoes (Diptera: Culicidae) in northwestern Iran by using rDNA-ITS2. Japanese Journal of Infectious Diseases. 2016;69(4):319-22.

126. Nikookar SH, Azari-Hamidian S, Fazeli-Dinan M, Nasab SN, Aarabi M, Ziapour SP, et al. Species composition, co-occurrence, association and affinity indices of mosquito larvae (Diptera: Culicidae) in Mazandaran Province, northern Iran. Acta Trop. 2016;157:20-9.

127. Omrani SM, Moosavi SF, Manouchehri K. Microsporidium infecting Anopheles supepictus (Diptera: Culicidae) larvae. Journal of Arthropod-Borne Diseases. 2016;10(3):415-22.

128. Sofizadeh A, Edalat H, Abai MR, Hanafi-Bojd AA. Fauna and some biological characteristics of Anopheles mosquitoes (Diptera: Culicidae) in Kalaleh County, Golestan Province, northeast of Iran. Asian Pacific Journal of Tropical Biomedicine. 2016;6(9):730-4.

129. Soltani Z, Keshavarzi D. Species composition of mosquitoes (Diptera: Culicidae) in Farashband district, southwest of Iran. Journal of Entomological and Acarological Research. 2016;48(3):372-7.

130. Yeryan M, Basseri HR, Hanafi-Bojd AA, Raeisi A, Edalat H, Safari R. Bio-ecology of malaria vectors in an endemic area, Southeast of Iran. Asian Pacific Journal of Tropical Medicine. 2016;9(1):32-8.

131. Keshavarzi D, Soltani Z, Ebrahimi M, Soltani A, Nutifafa GG, Soltani F, et al. Monthly prevalence and diversity of mosquitoes (Diptera: Culicidae) in Fars Province, Southern Iran. Asian Pacific Journal of Tropical Disease. 2017;7(2):112-20.

132. Nikookar SH, Fazeli-Dinan M, Azari-Hamidian S, Mousavinasab SN, Aarabi M, Ziapour SP, et al. Correlation between mosquito larval density and their habitat physicochemical characteristics in Mazandaran Province, northern Iran. PLoS Neglected Tropical Diseases. 2017;11(8).

133. Omrani SM, Moosavi SF, Farrokhi E. Parathelohania iranica sp. nov. (Microsporidia: Amblyosporidae) infecting malaria mosquito Anopheles superpictus (Diptera: Culicidae): Ultrastructure and molecular characterization. Journal of Invertebrate Pathology. 2017;146:1-6.

134. Soltani Z, Keshavarzi D, Ebrahimi M, Soltani A, Moemenbellah-Fard MJ, Soltani F, et al. The fauna and active season of mosquitoes in west of Fars province, southwest of Iran. Archives of Razi Institute. 2017;72(3):203-8.

135. Yazdi F, Nikookar SH, Fazeli-Dinan M, Hosseini SA, Yazdi M, Ziapour SP, et al. Diversity and species composition of mosquitoes (Culicidae: Diptera) in Noor County, northern Iran. Tropical Biomedicine. 2017;34(1):14-21.

136. Azari-Hamidian S, Norouzi B, Noorallahi A, Hanafi-Bojd AA. Seasonal activity of adult mosquitoes (Diptera: Culicidae) in a focus of dirofilariasis and west nile infection in Northern Iran. Journal of Arthropod-Borne Diseases. 2018;12(4):398-413.

137. Gorouhi MA, Oshaghi MA, Vatandoost H, Enayati AA, Abai MR, Karami M, et al. Biochemical Basis of Cyfluthrin and DDT Resistance in Anopheles stephensi (Diptera: Culicidae) in malarious area of Iran. Journal of Arthropod-Borne Diseases. 2018;12(3):310-20.

138. Nejati J, Saghafipour A, Vatandoost H, Moosa-Kazemi SH, Haghi AM, Sanei-Dehkordi A. Bionomics of <i>Anopheles subpictus</i> (Diptera: Culicidae) in a Malaria Endemic Area, Southeastern Iran. Journal of Medical Entomology. 2018;55(5):1182-7.

139. Nikookar SH, Fazeli-Dinan M, Azari-Hamidian S, Nasab SNM, Aarabi M, Ziapour SP, et al. Fauna, ecological characteristics, and checklist of the mosquitoes in mazandaran province, northern Iran. Journal of Medical Entomology. 2018;55(3):1-12.

140. Sofizadeh A, Shoraka H, Mesgarian F, Ozbaki GM, Gharaninia A, Sahneh E, et al. Fauna and larval habitats characteristics of mosquitoes (diptera: Culicidae) in Golestan Province, Northeast of Iran, 2014-2015. Journal of Arthropod-Borne Diseases. 2018;12(3):240-51.

141. Asadi Saatlou Z, Sedaghat MM, Taghilou B, Gholizadeh S. Identification of novel Glutathione S-Transferases epsilon 2 mutation in Anopheles maculipennis s.s. (Diptera: Culicidae). Heliyon. 2019;5(8).

142. Gholami S, Bakhshi H, Moosa-Kazemi SH, Zahraei-Ramazani A, Chavshin A, Sedaghat MM. Molecular characterization of Anopheles sacharovi based on sequences of ITS2-rDNA region and COI gene in North of Iran. Journal of Arthropod-Borne Diseases. 2019;13(2):135-44.

143. Karami M, Saboori A, Asadi M, Moosa-Kazemi SH, Gorouhi MA, Haghi FM, et al. Parasitism of mosquitoes (Diptera: Culicidae) by water mite larvae (Acari: Hydrachnidia) in Amol, Mazandaran Province, northern Iran. Systematic and Applied Acarology. 2019;24(3):423-34.

144. Paksa A, Sedaghat MM, Vatandoost H, Yaghoobi-Ershadi MR, Moosa-Kazemi SH, Hazratian T, et al. Biodiversity of mosquitoes (Diptera: Culicidae) with emphasis on potential arbovirus vectors in East Azerbaijan province, northwestern Iran. Journal of Arthropod-Borne Diseases. 2019;13(1):62-75.

145. Edalat H, Mahmoudi M, Sedaghat MM, Moosa-Kazemi SH, Kheirandish S. Ecology of malaria vectors in an endemic area, Southeast of Iran. Journal of Arthropod-Borne Diseases. 2020;14(4):325-43.

146. Ghavami MB, Khoeini S, Djadid ND. Molecular characteristics of odorant-binding protein 1 in Anopheles maculipennis. Malaria Journal. 2020;19(1).

147. Khoobdel M, Keshavarzi D, Sobati H, Akbari M. Species diversity, habitat and abundance of culicid mosquitoes in bushehr province, south of Iran. Biodiversitas. 2020;21(4):1401-6.

148. Mojahedi AR, Safari R, Yarian M, Pakari A, Raeisi A, Edalat H, et al. Biting and resting behaviour of malaria vectors in bandar-abbas county, islamic republic of iran. Eastern Mediterranean Health Journal. 2020;26(10):1218-26.

149. Asgarian TS, Moosa-Kazemi SH, Sedaghat MM, Dehghani R, Yaghoobi-Ershadi MR. Fauna and larval habitat characteristics of mosquitoes (Diptera: Culicidae) in Kashan County, Central Iran, 2019. Journal of Arthropod-Borne Diseases. 2021;15(1):69-81.

150. Faraji-Fard P, Ahmadi-Angali K, Behbahani A. Species Variety of the Calf and Human-Attracted Mosquitoes in Southwest Iran. Journal of Arthropod-Borne Diseases. 2021;15(2):162-70.

151. Kassiri H, Dehghani R, Khodkar I, Moosa-Kazemi SH, Asgarian TS, Golafshan AH, et al. Determination of fauna and abundance of larvae of the mosquitoes (Diptera: Culicidae) in paddy fields in Lenjan and Mobarakeh Counties, Isfahan Province, Center of Iran. Journal of Entomological Research. 2021;45(4):610-4.

152. Mirahmadi H, Rahmati-Balaghaleh M, Etemadi S, Khabisi SA, Tabatabaei SM, Mehravaran A, et al. Molecular detection of common plasmodium species in malaria vectors in villages of chabahar and konarak, iran. Shiraz E Medical Journal. 2021;22(7).

153. Moosa-Kazemi SH, Asgarian TS, Sedaghat MM, Javar S. Pathogenic fungi infection attributes of malarial vectors Anopheles maculipennis and Anopheles superpictus in central Iran. Malaria Journal. 2021;20(1).

154. Moosa-Kazemi SH, Etemadi Y, Sedaghat MM, Vatandoost H, Mokhayeri H, Kayedi MH. Investigation on Mosquitoes Fauna (Diptera: Culicidae) and Probable Vector of West Nile Virus in Lorestan Province, Western Iran. Journal of Arthropod-Borne Diseases. 2021;15(4):397-404.

155. Soltani A, Hoseini Z, Azizi K, Alipour H. A faunal study on medically important mosquitoes (Diptera: Culicidae) in Qir and Karzin from Fars province, southern Iran, during 2017-18. Journal of Entomological and Acarological Research. 2021;53(2):1-7.

156. Sharifi F, Banafshi O, Rasouli A, Ghoreishi S, Saeedi S, Khalesi M, et al. Biodiversity and Spatial Distribution of Mosquitoes (Diptera: Culicidae) in Kurdistan Province, Western Iran. J Arthropod Borne Dis. 2022;16(4):350-63.

157. Amirkafi A, Madjdzadeh SM, Gorouhi MA, Sanei-Dehkordi A, Alizadeh I, Afshar AA. Species Diversity and Distribution Pattern of Anopheline Mosquitoes (Diptera: Culicidae) in Qaleh Ganj County, Southeast of Iran. Journal of Health Sciences and Surveillance System. 2023;11(3):464-71.

158. Hajibeygi R, Hejripour SZ, Taghavi N, Shahali H, Zarei S, Nouri M, et al. Evaluation of the knockdown resistance locus (kdr) in Anopheles stephensi (Diptera: Culicidae) in southeastern Iran. Journal of Vector Borne Diseases. 2023;60(4):444-8.

159. Nasiri Z, Hosseinizadeh ZS, Sayyadi Z, Alipour H. Entomological survey of malaria vectors in Dashtestan County, South of Iran. Journal of Parasitic Diseases. 2023;47(1):161-6.

160. Askari F, Paksa A, Shahabi S, Saeedi S, Sofizadeh A, Vahedi M, et al. Population genetic structure and phylogenetic analysis of <i>Anopheles hyrcanus</i> (Diptera: Culicidae) inferred from DNA sequences of nuclear ITS2 and the mitochondrial COI gene in the northern part of Iran. Bmc Infectious Diseases. 2024;24(1).

161. Etherington D, Sellick G. Notes on the bionomics of anopheles sacharovi in Persia and Iraq. Bulletin of Entomological Research. 1946;37(2):191-5.

162. Pringle G. The Identification of the Larvae of Anopheles stephensi Liston and Anopheles superpictus Grassi in Iraq. Bulletin of Entomological Research. 1952;42(4):779-83.

163. Gramiccia G, de Meillon B, Petrides J, Ulrich AM. Resistance to DDT in Anopheles stephensi in southern Iraq. Bull World Health Organ. 1958;19(6):1102-4.

164. Abul-Hab J. Malaria vector survey in North Iraq. I. Provinces of Naynawah and Dhook. Bulletin of endemic diseases. 1969;11(1):117-33.

165. Niazi A, Hat Hat YAK. Antilarval activities in Iraq. BULLENDEMDIS. 1973;14(1-4):61-70.

166. Al-Ghoury AA, El-Hashimi WK, Abul-Hab J. Epidemiology of malaria and predictions of retransmission in Babylon governorate, Iraq. Eastern Mediterranean Health Journal. 2006;12(3-4):270-9.

167. Hantosh HA, Hassan HM, Ahma B, Al-fatlawy A. Mosquito species geographical distribution in Iraq 2009. Journal of Vector Borne Diseases. 2012;49(1):33-5.

168. Farid MA. Ineffectiveness of DDT residual spraying in stopping malaria transmission in the Jordan Valley. Bull World Health Organ. 1954;11(4-5):765-83.

169. Amr ZS, Al-Khalili Y, Arbaji A. Larval mosquitoes collected from northern Jordan and the Jordan Valley. Journal of the American Mosquito Control Association. 1997;13(4):375-8.

170. Cropper J. The geographical distribution of anopheles and malarial fever in upper palestine. Journal of Hygiene. 1902;2(1):47-57.

171. Garrett-Jones C, Gramiccia G. Evidence of the development of resistance to DDT by Anopheles sacharovi in the Levant. Bulletin of the World Health Organization. 1954;11(4-5):865-83.

172. Knio KM, Markarian N, Kassis A, Nuwayri-Salti N. A two-year survey on mosquitoes of Lebanon. Parasite. 2005;12(3):229-35.

173. Bailly Choumara H. Preliminary study of a catch of Anopheles labranchiae by CDC light traps in the region of Larache, Morocco. Bulletin of the World Health Organization. 1973;49(1):49-55.

174. Deschiens R, Cornu M. Epidemiologic and parasitologic survey on Hassan Addakhil barrage and its harnessing in Tafilalet (Morocco, May/June 1975). Bulletin de la Societe de Pathologie Exotique et de ses Filiales. 1975;68(5):482-91.

175. Faraj C, Adlaoui E, Saaf N, Romi R, Boccolini D, Di Luca M, et al. Note on Anopheles maculipennis complex in Morocco. Bulletin de la Societe de Pathologie Exotique. 2004;97(4):293-4.

176. Faraj C, Adlaoui E, Brengues C, Fontenille D, Lyagoubi M. Resistance of Anopheles labranchiae to DDT in Morocco: Identification of the mechanisms and choice of replacement insecticide. Eastern Mediterranean Health Journal. 2008;14(4):776-83.

177. Faraj C, Adlaoui E, Ouahabi S, Lakraa E, Elkohli M, El Aouad R. Extension to the north of the distribution area of Anopheles (Cellia) d'thali Patton, 1905. Bulletin de la Societe de Pathologie Exotique. 2008;101(1):62-4.

178. Faraj C, Adlaoui E, Ouahabi S, Rhajaoui M, Fontenille D, Lyagoubi M. Entomological investigations in the region of the last malaria focus in Morocco. Acta Tropica. 2009;109(1):70-3.

179. Majda L, Chafika F, Abderrahim S, Mustapha A, Mohamed R, Rajae E. ECOLOGY AND SPATIAL DISTRIBUTION OF BREEDING SITES OF <i>ANOPHELES</i> LARVAE IN LARACHE PROVINCE, MOROCCO. American Journal of Tropical Medicine and Hygiene. 2010;83(5):61-2.

180. Adlaoui E, Faraj C, El Bouhmi M, El Aboudi A, Ouahabi S, Tran A, et al. Mapping malaria transmission risk in northern morocco using entomological and environmental data. Malaria research and treatment. 2011;2011:391463.

181. Laboudi M, Faraj C, Sadak A, Harrat Z, Boubidi SC, Harbach RE, et al. DNA barcodes confirm the presence of a single member of the Anopheles maculipennis group in Morocco and Algeria: An. sicaulti is conspecific with An. labranchiae. Acta Tropica. 2011;118(1):6-13.

182. Laboudi M, Faraj C, Sadak A, Azelmate M, Rhajaoui M, El-Aouad R. Some environmental factors associated with anopheles labranchiae larval distribution during summer 2009, in Larache Province, Morocco. African Entomology. 2012;20(2):229-38.

183. El-Akhal F, Guemmouh R, Maniar S, Taghzouti K, El Ouali Lalami A. Larvicidal activity of essential oils of thymus vulgaris and origanum majorana (Lamiaceae) against of the malaria vector anopheles labranchiae (diptera: Culicidae). International Journal of Pharmacy and Pharmaceutical Sciences. 2016;8(3):372-6.

184. Filali Mouatassem T, Faraj C, Guemmouh R, Rais N, El Ouali Lalami A. Quantitative inventory of mosquito larvae (Diptera: Culicidae) and physicochemical analysis of aquatic habitats in the region of Fez, Morocco. Bulletin de la Societe de Pathologie Exotique. 2019;112(2):105-13.

185. Mouatassem TF, Lalami AEO, Faraj C, Rais N, Guemmouh R. [Culicidae Larvae and Their Seasonal Dynamics in the Region of Fez-Meknes, Morocco]. Medecine tropicale et sante internationale. 2021;1(2).

186. Filali Mouatassem T, Faraj C, Guemmouh R, Fadil M, Rais N, El Asmi H, et al. Biotypology of Culicidian Species in the Region of Fez, Central Morocco Using the Statistical Analytical Methods. Tropical Journal of Natural Product Research. 2024;8(5):7172-80.

187. Roberts D. Mosquitoes (Diptera:Culicidae) breeding in brackish water: female ovipositional preferences or larval survival? J Med Entomol. 1996;33(4):525-30.

188. Baomar AT, Mohamed AG. Malaria outbreak in a malaria-free region in Oman 1998: Unknown impact of civil war in Africa. Public Health. 2000;114(6):480-3.

189. Carmichael GT. Anopheline control through water management. The American journal of tropical medicine and hygiene. 1972;21(5):782-6.

190. Akiyama J, editor Anopheles mosquito studies in Pakistan using outlet and inlet traps. Transactions of the Royal Society of Tropical Medicine and Hygiene; 1974.

191. Reisen WK, Aslamkhan M. Observations on the swarming and mating behaviour of Anopheles culicifacies Giles in nature. Bulletin of the World Health Organization. 1976;54(2):155-8.

192. Reisen WK. A quantitative mosquito survey on 7 villages in Punjab province, Pakistan with notes on bionomics, sampling methodology and the effect of insecticides. Southeast Asian Journal of Tropical Medicine and Public Health. 1978;9(4):587-601.

193. Reisen WK, Aslamkhan M. Biting rhythms of some Pakistan mosquitoes (Diptera: Culicidae). Bulletin of Entomological Research. 1978;68(2):313-30.

194. Reisen WK, Mahmood F, Parveen T. Anopheles subpictus Grassi: Observations on survivorship and population size using mark-release-recapture and dissection methods. Researches on Population Ecology. 1979;21(1):12-29.

195. Aslamkhan M, Pervez SD. Imported filariasis in pakistan. Transactions of the Royal Society of Tropical Medicine and Hygiene. 1981;75(6):869-71.

196. Hemingway J. The biochemical nature of malathion resistance in Anopheles stephensi from Pakistan. Pesticide Biochemistry and Physiology. 1982;17(2):149-55.

197. Reisen WK, Azra K, Mahmood F. Anopheles culicifacies (Diptera: Culicidae): horizontal and vertical estimates of immature development and survivorship in rural Punjab province, Pakistan. Journal of Medical Entomology. 1982;19(4):413-22.

198. Reisen WK, Mahmood F, Parveen T. Seasonal trends in population size and survivorship of Anopheles culicifacies, An. stephensi and An. subpictus (Diptera: Culicidae) in rural Punjab province, Pakistan. Journal of medical entomology. 1982;19(1):86-97.

199. Mahmood F, Sakai RK. Inversion polymorphisms in natural populations of Anopheles stephensi. Canadian journal of genetics and cytology Journal canadien de génétique et de cytologie. 1984;26(5):538-46.

200. Mahmood F, Sakai RK, Akhtar K. Vector incrimination studies and observations on species A and B of the taxon Anopheles culicifacies in Pakistan. Transactions of the Royal Society of Tropical Medicine and Hygiene. 1984;78(5):607-16.

201. Nalin DR, Mahood F, Rathor H, Muttalib A, Sakai R, Chowdhry MA, et al. A point survey of periurban and urban malaria in Karachi. Journal of Tropical Medicine and Hygiene. 1985;88(1):7-15.

202. Hewitt S, Kamal M, Muhammad N, Rowland M. An entomological investigation of the likely impact of cattle ownership on malaria in an Afghan refugee camp in the North West Frontier Province of Pakistan. Medical and Veterinary Entomology. 1994;8(2):160-4.

203. Hewitt S, Rowland M, Muhammad N, Kamal M, Kemp E. Pyrethroid‐sprayed tents for malaria control: an entomological evaluation in Pakistan. Medical and Veterinary Entomology. 1995;9(4):344-52.

204. Bouma MJ, Parvez SD, Nesbit R, Winkler AM. Malaria control using permethrin applied to tents of nomadic Afghan refugees in northern Pakistan. Bull World Health Organ. 1996;74(4):413-21.

205. Rowland M, Durrani N, Kenward M, Mohammed N, Urahman H, Hewitt S. Control of malaria in Pakistan by applying deltamethrin insecticide to cattle: A community-randomised trial. Lancet. 2001;357(9271):1837-41.

206. Graham K, Mohammad N, Rehman H, Farhan M, Kamal M, Rowland M. Comparison of three pyrethroid treatments of top-sheets for malaria control in emergencies: Entomological and user acceptance studies in an afghan refugee camp in pakistan. Medical and Veterinary Entomology. 2002;16(2):199-206.

207. Mukhtar M, Herrel N, Amerasinghe FP, Ensink J, Van Der Hoek W, Konradsen F. Role of wastewater irrigation in mosquito breeding in south Punjab, Pakistan. Southeast Asian Journal of Tropical Medicine and Public Health. 2003;34(1):72-80.

208. Yasinzai MI, Kakarsulemankhel JK. A study of prevalence of malaria infection in urban areas of District Quetta, Pakistan. Pakistan Journal of Zoology. 2004;36(1):75-9.

209. Mukhtar M, Ensink J, Van Der Hoek W, Amerasinghe FP, Konradsen F. Importance of waste stabilization ponds and wastewater irrigation in the generation of vector mosquitoes in Pakistan. Journal of Medical Entomology. 2006;43(5):996-1003.

210. Ali N, Hume JC, Dadzie SK, Donnelly MJ. Molecular genetic studies of Anopheles stephensi in Pakistan. Med Vet Entomol. 2007;21(3):265-9.

211. Ali N, Khan K, Kausar A. Study on mosquitoes of Swat Ranizai Sub Division of Malakand. Pakistan Journal of Zoology. 2013;45(2):503-10.

212. Khan SY, Butt A, Tahir HM, Shafaat S. Response of anopheles stephensi to deltamethrin in Lahore District, Pakistan. Asian Journal of Chemistry. 2013;25(1):165-9.

213. Naz S, Maqbool A, Ahmad MUD, Anjum AA, Zaman S. Efficacy of ivermectin for control of zoophilic malaria vectors in pakistan. Pakistan Journal of Zoology. 2013;45(6):1585-91.

214. Rathor HR, Nadeem G, Khan IA. Pesticide susceptibility status of anopheles mosquitoes in four flood-affected districts of South Punjab, Pakistan. Vector-Borne and Zoonotic Diseases. 2013;13(1):60-6.

215. Qasim M, Naeem M, Bodlah I. Mosquito (Diptera: Culicidae) of murree hills, Punjab, Pakistan. Pakistan Journal of Zoology. 2014;46(2):523-9.

216. Rana SM, Khan EA, Yaqoob A, Latif AA, Abbasi MM. Susceptibility and irritability of adult forms of main malaria vectors against insecticides used in the indoor residual sprays in muzaffargarh district, Pakistan: A field survey. Journal of Medical Entomology. 2014;51(2):387-91.

217. Shaikh S, Kazmi SJH, Qureshi S. Monitoring the diversity of malaria and dengue vector in Karachi: studying variation of genera and subgenera of mosquitoes under different ecological conditions. Ecological Processes. 2014;3(1).

218. Ali N, Noreen S, Khan K, Wahid S. Population dynamics of mosquitoes and malaria vector incrimination in district Charsadda, Khyber Pakhtunkhwa (KP) Pakistan. Acta Tropica. 2015;141(Part A):25-31.

219. Bibi M, Zahoor MK, Zahoor MA, Ashraf HM, Majeed HN, Nasir S, et al. Genetic analysis of mosquitoes from rural and urban areas of Sialkot, Pakistan. International Journal of Agriculture and Biology. 2015;17(4):809-14.

220. Oneeb M, Maqbool A, Lateef M, Babar ME. Detection of Plasmodium falciparum infection in Anopheles stephensi in Punjab, Pakistan. Pakistan Journal of Zoology. 2015;47(4):1192-5.

221. Manzoor F, Shabbir R, Sana M, Nazir S, Khan MA. Determination of species composition of mosquitoes in Lahore, Pakistan. Journal of Arthropod-Borne Diseases. 2020;14(1):106-15.

222. Attaullah M, Gul S, Bibi D, Andaleeb A, Ilahi I, Siraj M, et al. Diversity, distribution and relative abundance of the mosquito fauna (Diptera: Culicidae) of Malakand and Dir Lower, Pakistan. Brazilian journal of biology = Revista brasleira de biologia. 2021;83:e247374.

223. Mehmood A, Naeem M, Raza ABM, Riaz MA, Majeed MZ, Khan N, et al. Species Distribution, Abundance and Diversity of Mosquitoes (Diptera: Culicidae) in District Jhelum (Punjab, Pakistan). Pakistan Journal of Agricultural Research. 2022;35(3):508-13.

224. Kardousha MM. Additional records of vector mosquito diversity collected from Al Khor district of North-eastern Qatar. Asian Pacific Journal of Tropical Disease. 2015;5(10):804-7.

225. Peffly RL. Insecticide resistance in anophelines in eastern Saudi Arabia. Bulletin of the World Health Organization. 1959;20:757-76.

226. Abdoon AMMO, Alshahrani AM. Prevalence and distribution of anopheline mosquitoes in malaria endemic areas of Asir region, Saudi Arabia. Eastern Mediterranean Health Journal. 2003;9(3):240-7.

227. Al Ahmed AM, Al Kuriji MA, Kheir SM, Al Zahrni AA. Distribution and Seasonal Abundance of Different Mosquito Species (Diptera: Culicidae) in Al Bahah Region, Saudi Arabia. Arab Gulf Journal of Scientific Research. 2010;28(2):67-78.

228. Ahmed AM, Shaalan EA, Aboul-Soud MA, Tripet F, Al-Khedhairy AA. Mosquito vectors survey in the AL-Ahsaa district of eastern Saudi Arabia. Journal of insect science (Online). 2011;11:176.

229. Al-Sheik AA. Larval habitat, ecology, seasonal abundance and vectorial role in malaria transmission of Anopheles arabiensis in Jazan Region of Saudi Arabia. Journal of the Egyptian Society of Parasitology. 2011;41(3):615-34.

230. Alahmed AM. Mosquito fauna (Diptera: Culicidae) of the Eastern Region of Saudi Arabia and their seasonal abundance. Journal of King Saud University - Science. 2012;24(1):55-62.

231. Khater EI, Sowilem MM, Sallam MF, Alahmed AM. Ecology and habitat characterization of mosquitoes in Saudi Arabia. Tropical Biomedicine. 2013;30(3):409-27.

232. Al Ashry HA, Kenawy MA, Shobrak M. Fauna of mosquito larvae (Diptera: Culicida) in Asir Provence, Kingdom of Saudi Arabia. Journal of the Egyptian Society of Parasitology. 2014;44(1):173-86.

233. Mahyoub JA, Al-Harbi OS, Al-Ghamdi KM, Mangoud AAH, Al-Solami HM. Population dynamics of different mosquito genera and species in Makkah city, Saudi Arabia. Bioscience Biotechnology Research Communications. 2015;8(2):116-25.

234. Alsheikh AA, Albarrak AA, Daffalla OM, Noureldin EM, Mohammed WS, Shrwani KJ, et al. THE USE OF NESTED-PCR TO DETECT THE PRESENCE OF PLASMODIUM IN ANOPHELES ARABIENSIS IN JAZAN REGION, SAUDI ARABIA. J Egypt Soc Parasitol. 2017;47(1):151-8.

235. Hassan MI, Kenawy MA, Al Ashry HA, Shobrak M. MOSQUITOES (DIPTERA: CULICIDAE) OF THE WESTERN COASTAL AREA, KINGDOM OF SAUDI ARABIA: SPECIES COMPOSITION, ABUNDANCE, DIVERSITY AND MEDICAL IMPORTANCE. J Egypt Soc Parasitol. 2017;47(1):167-76.

236. Shaalan EAS, Abdelsalam S, Elmenshawy O, Al-Kahtani MA. Mosquito vectors survey reveals new record of Culiseta subochrea in Al-Ahsa Oasis, Saudi Arabia. Asian Pacific Journal of Tropical Disease. 2017;7(2):106-11.

237. Munawar K, Saleh A, Afzal M, Qasim M, Khan KA, Zafar MI, et al. Molecular characterization and phylogenetic analysis of anopheline (Anophelinae: Culicidae) mosquitoes of the Oriental and Afrotropical Zoogeographic zones in Saudi Arabia. Acta Tropica. 2020;207.

238. Alghamdi TS, Zahrani MRA, Gharsan FN, Ghamdi KMA, Mahyoub JA. Identification of mosquito species and determination of population density in the Taif governorate, Saudi Arabia. Journal of Entomological and Acarological Research. 2021;53(1):1-6.

239. Ali S, Samake JN, Spear J, Carter TE. Morphological identification and genetic characterization of <i>Anopheles stephensi</i> in Somaliland. Parasites & Vectors. 2022;15(1).

240. Lewis DJ. A NEW SPECIES OF ANOPHELES FEOM THE ANGLO‐EGYPTIAN SUDAN. Proceedings of the Royal Entomological Society of London Series B, Taxonomy. 1937;6(9):181-3.

241. Omer SM, Cloudsley-Thompson JL. Dry season biology of anopheles gambiae giles in the sudan. Nature. 1968;217(5131):879-80.

242. Haridi AM. Partial exophily of Anopheles gambiae species B in the Khashm Elgirba area in eastern Sudan. Bulletin of the World Health Organization. 1972;46(1):39-46.

243. Akiyama J. Exophily in Anopheles gambiae species B in the Sudan. Transactions of the Royal Society of Tropical Medicine and Hygiene. 1973;67(4):440.

244. Hemingway J. Biochemical studies on malathion resistance in Anopheles arabiensis from Sudan. Transactions of the Royal Society of Tropical Medicine and Hygiene. 1983;77(4):477-80.

245. Mahmoud AA. Mosquitofish Gambusia affinis holbrooki as a malaria vector control agent in Gezira irrigation canals of the Sudan. Journal of the American Mosquito Control Association. 1985;1(4):524-6.

246. Dukeen MYH, Omer SM. Ecology of the malaria vector Anopheles arabiensis Patton (Diptera: Culicidae) by the nile in northern Sudan. Bulletin of Entomological Research. 1986;76(3):451-67.

247. El Sayed BB, Arnot DE, Mukhtar MM, Baraka OZ, Dafalla AA, Elnaiem DE, et al. A study of the urban malaria transmission problem in Khartoum. Acta Trop. 2000;75(2):163-71.

248. Petrarca V, Nugud AD, Ahmed MA, Haridi AM, Di Deco MA, Coluzzi M. Cytogenetics of the Anopheles gambiae complex in Sudan, with special reference to An. arabiensis: relationships with East and West African populations. Med Vet Entomol. 2000;14(2):149-64.

249. Hamad AA, Nugud AED, Arnot DE, Giha HA, Abdel-Muhsin AMA, Satti GMH, et al. A marked seasonality of malaria transmsission in two rural sites in eastern Sudan. Acta Tropica. 2002;83(1):71-82.

250. Himeidan YE, Dukeen MY, El-Rayah el A, Adam I. Anopheles arabiensis: abundance and insecticide resistance in an irrigated area of eastern Sudan. Eastern Mediterranean health journal = La revue de sante de la Mediterranee orientale = al-Majallah al-sihhiyah li-sharq al-mutawassit. 2004;10(1-2):167-74.

251. Himeidan YE, Chen H, Chandre F, Donnelly MJ, Yan G. Short report: permethrin and DDT resistance in the malaria vector Anopheles arabiensis from eastern Sudan. Am J Trop Med Hyg. 2007;77(6):1066-8.

252. Matambo TS, Abdalla H, Brooke BD, Koekemoer LL, Mnzava A, Hunt RH, et al. Insecticide resistance in the malarial mosquito Anopheles arabiensis and association with the kdr mutation. Medical and Veterinary Entomology. 2007;21(1):97-102.

253. Abdalla H, Matambo TS, Koekemoer LL, Mnzava AP, Hunt RH, Coetzee M. Insecticide susceptibility and vector status of natural populations of Anopheles arabiensis from Sudan. Transactions of the Royal Society of Tropical Medicine and Hygiene. 2008;102(3):263-71.

254. Bashir A, Abu Hassan BA, Che Salmah MR, Rahman WA. Efficacy of AGNIQUE® (MMF) monomolecular surface film against immature stages of Anopheles arabiensis patton and Culex SPP (Diptera: Culicidae) in Khartoum, Sudan. Southeast Asian Journal of Tropical Medicine and Public Health. 2008;39(2):222-8.

255. Helinski ME, Hassan MM, El-Motasim WM, Malcolm CA, Knols BG, El-Sayed B. Towards a sterile insect technique field release of Anopheles arabiensis mosquitoes in Sudan: irradiation, transportation, and field cage experimentation. Malar J. 2008;7:65.

256. Himeidan YE, El Rayah AE. Role of some environmental factors on the breeding activity of Anopheles arabiensis in New Halfa town, eastern Sudan. Eastern Mediterranean health journal = La revue de sante de la Mediterranee orientale = al-Majallah al-sihhiyah li-sharq al-mutawassit. 2008;14(2):252-9.

257. Ageep TB, Cox J, Hassan MM, Knols BG, Benedict MQ, Malcolm CA, et al. Spatial and temporal distribution of the malaria mosquito Anopheles arabiensis in northern Sudan: Influence of environmental factors and implications for vector control. Malaria Journal. 2009;8(1).

258. Elimam AM, Elmalik KH, Ali AS. Larvicidal, adult emergence inhibition and oviposition deterrent effects of foliage extract from Ricinus communis L. against Anopheles arabiensis and Culex quinquefasciatus in Sudan. Tropical Biomedicine. 2009;26(2):130-9.

259. Ranson H, Abdallah H, Badolo A, Guelbeogo WM, Kerah-Hinzoumbé C, Yangalbé-Kalnoné E, et al. Insecticide resistance in Anopheles gambiae: Data from the first year of a multi-country study highlight the extent of the problem. Malaria Journal. 2009;8(1).

260. Himeidan YE, Elzaki MM, Kweka EJ, Ibrahim M, Elhassan IM. Pattern of malaria transmission along the Rahad River basin, Eastern Sudan. Parasites and Vectors. 2011;4(1).

261. Himeidan YE, Muzamil H, Jones CM, Ranson H. Extensive permethrin and DDT resistance in Anopheles arabiensis from eastern and central Sudan. Parasites and Vectors. 2011;4(1).

262. Seidahmed OM, Abdelmajed MA, Mustafa MS, Mnzava AP. Insecticide susceptibility status of the malaria vector Anopheles arabiensis in Khartoum city, Sudan: differences between urban and periurban areas. Eastern Mediterranean health journal = La revue de sante de la Mediterranee orientale = al-Majallah al-sihhiyah li-sharq al-mutawassit. 2012;18(7):769-76.

263. Abuelmaali SA, Elaagip AH, Basheer MA, Frah EA, Ahmed FT, Elhaj HF, et al. Impacts of agricultural practices on insecticide resistance in the malaria vector Anopheles arabiensis in Khartoum State, Sudan. PLoS One. 2013;8(11):e80549.

264. Aboud M, Makhawi A, Verardi A, El Raba'a F, Elnaiem DE, Townson H. A genotypically distinct, melanic variant of Anopheles arabiensis in Sudan is associated with arid environments. Malaria Journal. 2014;13(1).

265. Hamza AM, El Rayah el A. A Qualitative Evidence of the Breeding Sites of Anopheles arabiensis Patton (Diptera: Culicidae) in and Around Kassala Town, Eastern Sudan. International journal of insect science. 2016;8:65-70.

266. Azrag RS, Mohammed BH. Anopheles arabiensis in Sudan: A noticeable tolerance to urban polluted larval habitats associated with resistance to Temephos. Malaria Journal. 2018;17(1).

267. Ismail BA, Kafy HT, Sulieman JE, Subramaniam K, Thomas B, Mnzava A, et al. Temporal and spatial trends in insecticide resistance in <i>Anopheles arabiensis</i> in Sudan: outcomes from an evaluation of implications of insecticide resistance for malaria vector control. Parasites & Vectors. 2018;11.

268. Korti MY, Ageep TB, Adam AI, Shitta KB, Hassan AA, Algadam AA, et al. Status of insecticide susceptibility in Anopheles arabiensis and detection of the knockdown resistance mutation (kdr) concerning agricultural practices from Northern Sudan state, Sudan. Journal of Genetic Engineering and Biotechnology. 2021;19(1).

269. Hamza AM, Elboshra SH. Mitotic metaphase karyotype of the mosquito Anopheles arabiensis Patton (Diptera: Culicidae) from Kassala State, eastern Sudan. Caryologia. 2023;76(2):15-21.

270. Onori E. Experience with mass drug administration as a supplementary attack measure in areas of vivax malaria. Bulletin of the World Health Organization. 1972;47(5):543-8.

271. Krida G, Bouattour A, Rhaim A, el Kebir A, Jlidi R. Preliminary investigation of four anopheles larvae samples susceptibility to chlorpyrifos in Tunisia. Archives de l'Institut Pasteur de Tunis. 1998;75(3-4):199-203.

272. Tabbabi A, Boussés P, Rhim A, Brengues C, Daaboub J, Ben-Alaya-Bouafif N, et al. Larval habitats characterization and species composition of anopheles mosquitoes in Tunisia, with particular attention to anopheles maculipennis complex. American Journal of Tropical Medicine and Hygiene. 2015;92(3):653-9.

273. Maffi M. On some larvae of the Myzomyia series collected in the Yemen. Parassitologia. 1971;13(3):449-54.

274. Kravchenko VK. Some aspects of the epidemiology of malaria in the People's Democratic Republic of Yemen. Meditsinskaya Parazitologiya i Parazitarnye Bolezni. 1979;48(4):10-3.

275. Al-Maktari MT, Bassiouny HK. Bionomics of anopheline vectors in Zabid District, Al-Hodeidah Governorate, Republic of Yemen. Eastern Mediterranean Health Journal. 1999;5(4):698-705.

276. Allan R, Weetman D, Sauskojus H, Budge S, Hawail TB, Baheshm Y. Confirmation of the presence of Anopheles stephensi among internally displaced people’s camps and host communities in Aden city, Yemen. Malaria Journal. 2023;22(1).
